# An evaluation methodology for machine learning-based tandem mass spectra similarity prediction

**DOI:** 10.1186/s12859-025-06194-1

**Published:** 2025-07-11

**Authors:** Michael Strobel, Alberto Gil-de-la-Fuente, Mohammad Reza Zare Shahneh, Yasin El Abiead, Roman Bushuiev, Anton Bushuiev, Tomáš Pluskal, Mingxun Wang

**Affiliations:** 1 Department of Computer Science and Engineering, University of California Riverside, 900 University Ave., Riverside, CA 92521 USA; 2 Information Technologies Department, Escuela Politécnica Superior, Universidad San Pablo-CEU, CEU Universities, Urbanización Montepríncipe, Boadilla Del monte, 28668 Madrid, Spain; 3 Skaggs School of Pharmacy and Pharmaceutical Science, University of California San Diego, 9255 Pharmacy Ln, San Diego, CA 92093 USA; 4 Institute of Organic Chemistry and Biochemistry, Czech Academy of Sciences, Flemingovo nám. 542/2, Prague, 16000 Czech Republic; 5 Czech Institute of Informatics, Robotics and Cybernetics, Jugoslávských partyzánů 1580/3, Prague, 16000 Czech Republic

**Keywords:** Mass spectrometry, Metabolomics, Spectral similarity measure, Machine learning, Benchmark

## Abstract

**Background:**

Untargeted tandem mass spectrometry serves as a scalable solution for the organization of small molecules. One of the most prevalent techniques for analyzing the acquired tandem mass spectrometry data (MS/MS) - called molecular networking - organizes and visualizes putatively structurally related compounds. However, a key bottleneck of this approach is the comparison of MS/MS spectra used to identify nearby structural neighbors. Machine learning (ML) approaches have emerged as a promising technique to predict structural similarity from MS/MS that may surpass the current state-of-the-art algorithmic methods. However, the comparison between these different ML methods remains a challenge because there is a lack of standardization to benchmark, evaluate, and compare MS/MS similarity methods, and there are no methods that address data leakage between training and test data in order to analyze model generalizability.

**Result:**

In this work, we present the creation of a new evaluation methodology using a train/test split that allows for the evaluation of machine learning models at varying degrees of structural similarity between training and test sets. We also introduce a training and evaluation framework that measures prediction accuracy on domain-inspired annotation and retrieval metrics designed to mirror real-world applications. We further show how two alternative training methods that leverage MS specific insights (e.g., similar instrumentation, collision energy, adduct) affect method performance and demonstrate the orthogonality of the proposed metrics. We especially highlight the role that collision energy plays in prediction errors. Finally, we release a continually updated version of our dataset online along with our data cleaning and splitting pipelines for community use.

**Conclusion:**

It is our hope that this benchmark will serve as the basis of development for future machine learning approaches in MS/MS similarity and facilitate comparison between models. We anticipate that the introduced set of evaluation metrics allows for a better reflection of practical performance.

**Supplementary Information:**

The online version contains supplementary material available at 10.1186/s12859-025-06194-1.

## Background

Tandem mass spectrometry (MS/MS) of small molecules is a cornerstone of the metabolomics and natural products fields. The high-throughput ability to collect MS/MS spectra has presented new discovery opportunities but also creates new challenges in data interpretation. A common approach to analyzing these MS/MS spectra is the matching of MS/MS against libraries of reference MS/MS spectra for the identification of exact and near structural analogues [1]. Additionally, the molecular networking paradigm [2] has arisen as a popular strategy to organize similar molecules even without compound annotation. The key underlying algorithmic process that enables these computational techniques is the ability to compare MS/MS spectra and determine a level of chemical structural similarity, even without identification. Algorithmic approaches such as cosine, modified cosine similarity, and spectral entropy [1, 3, 4] have been used to address this challenge in cases where the underlying structures are near-neighbors. Recently, algorithmic advances have expanded this approach to multiple modifications but rely on intermediary structures present in the data [5]. In addition, machine learning (ML) approaches have become viable alternatives that hold the promise of enhanced performance [6–9].

However, there are key limitations in the development and evaluation of these ML approaches that hinder their impact on the field. First, there are domain-knowledge barriers for new entrants to the field seeking to leverage existing data to build new models. Second, there is currently no standard dataset or proposed metrics for the community to effectively measure and compare the progress that is being made by new publications. Third, existing model’s ability to generalize to new molecules remains under-investigated. In this manuscript we aim to present a method that aims to address these issues for the MS/MS similarity problem.

First, we introduce a metadata harmonized, machine learning ready method and dataset for training and evaluating MS/MS spectrum similarity for machine learning. This training dataset draws from public MS/MS resources: GNPS [10] and MassBank [11]. We describe the benchmark’s creation and apply it to gain insight into existing models, including an exploration of out of distribution generalization, an active area of research in the parallel fields of computer vision and natural language processing [12–15]. Next, we highlight limitations in existing metrics and provide a set of evaluation metrics and reproducible tools that allow for the evaluation of future models in domain-relevant metrics. Finally, we build on the concept of living data in mass spectrometry [10, 16] and introduce infrastructure to periodically update these ML datasets.

## Results

### Training/test data preparation for structural similarity prediction

**Fig. 1:**
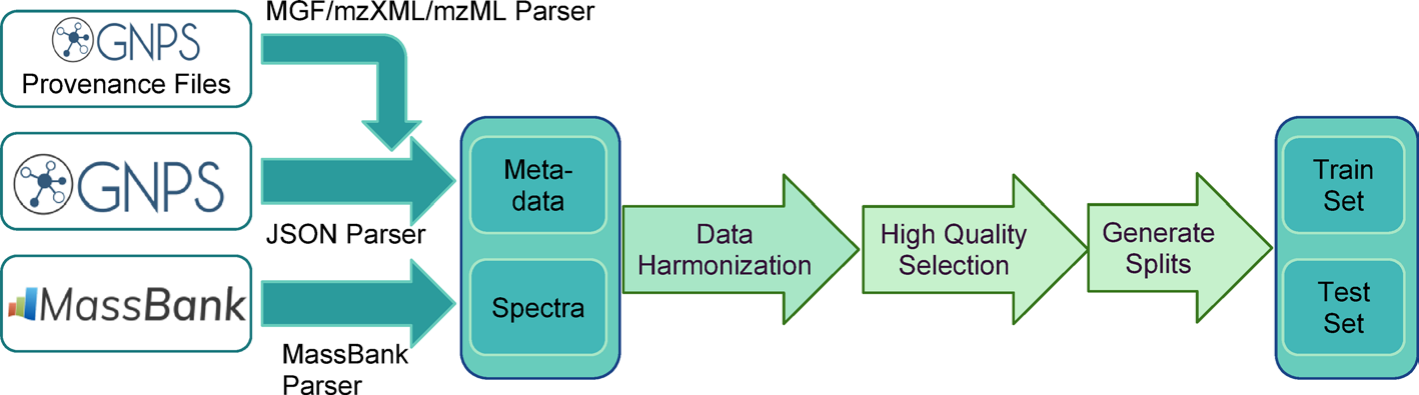
Schematic of data processing pipeline data used to generate our benchmark is collected from GNPS and MassBank EU. On top of existing metadata, provenance spectral files from GNPS are integrated into the main pipeline. Data from the two sources is first harmonized and cleaned (Methods: Dataset Processing & Cleaning), and then the highest quality annotations are selected and a conservative cleaning of spectra is performed (Methods: Selecting MS/MS Data for Machine Learning). Finally, data is split into training and test subsets ensuring low structural similarity between the molecules in different sets (Methods: Sampling Low Train-Test Similarity Pairs). Logos used in this figure are licensed under the Creative Commons Attribution 4.0 International License (see Availability of Data and Materials)

We collected and harmonized public data from the GNPS and MassBank spectral libraries, a schematic of the methodology can be seen in Fig. 1. As of July 2024, the input data contains 39,274 structures and 788,951 spectra. We harmonized MS/MS library metadata (including removing duplicate imported MS/MS) and canonicalized 2D structures leaving 37,363 structures and 699,317 spectra (see Methods: Dataset Processing & Cleaning). We further removed the GNPS MS/MS libraries with heterogeneous instrument acquisition (individual GNPS user contributions library) and libraries with a large number of unexplained MS/MS fragmentation (BMDMS), resulting in 459,250 spectra with 32,857 structures. We filtered to positive ion mode, leaving 29,702 structures and 292,810 spectra. Next, we filtered the MS/MS spectra to keep only those with a precursor *m/z* matching their annotations, and significant fragmentation in the MS/MS (see Methods: Selecting MS/MS Data for Machine Learning, SI Fig. 1). The final dataset contained 189,467 spectra from 28,132 structures. Daily updates of the harmonized-source data that continuously integrate user uploads are available online (See Availability of Data and Materials) and are available as quarterly Zenodo depositions.

We developed a method to create consistent train/test splits that emphasizes a more balanced coverage of relevant data space in two key dimensions: (1) similar and dissimilar structure pairs of MS/MS spectra (pairwise structure similarity diversity) and (2) similar and dissimilar structures between training and test (train-test similarity diversity) (See Methods: Sampling Low Train-Test Similarity Pairs). We define train-test similarity for a structure as the maximum similarity between a test structure, and all training examples. To adapt this definition to the pairwise nature of the problem, we use the mean train-test similarity for both structures in a test pair as the measure of train-test similarity. The resulting “balanced test set” is important for understanding how MS/MS similarity models generalize to new data and provides a comprehensive assessment of model performance across the possible and relevant input space.

The baseline methodology for constructing test/train splits is the random selection of unique chemical structures. For example, in the MS2DeepScore publication [8], 500 random unique structures (2,967 MS/MS spectra) were selected for the test. This random selection reflects the underlying distribution of structures, but under-samples regions that are of key interest for an MS/MS similarity model—specifically in lower train-test similarity and higher pairwise structure similarity (top left and middle of SI Fig. 1A).

**Fig. 2:**
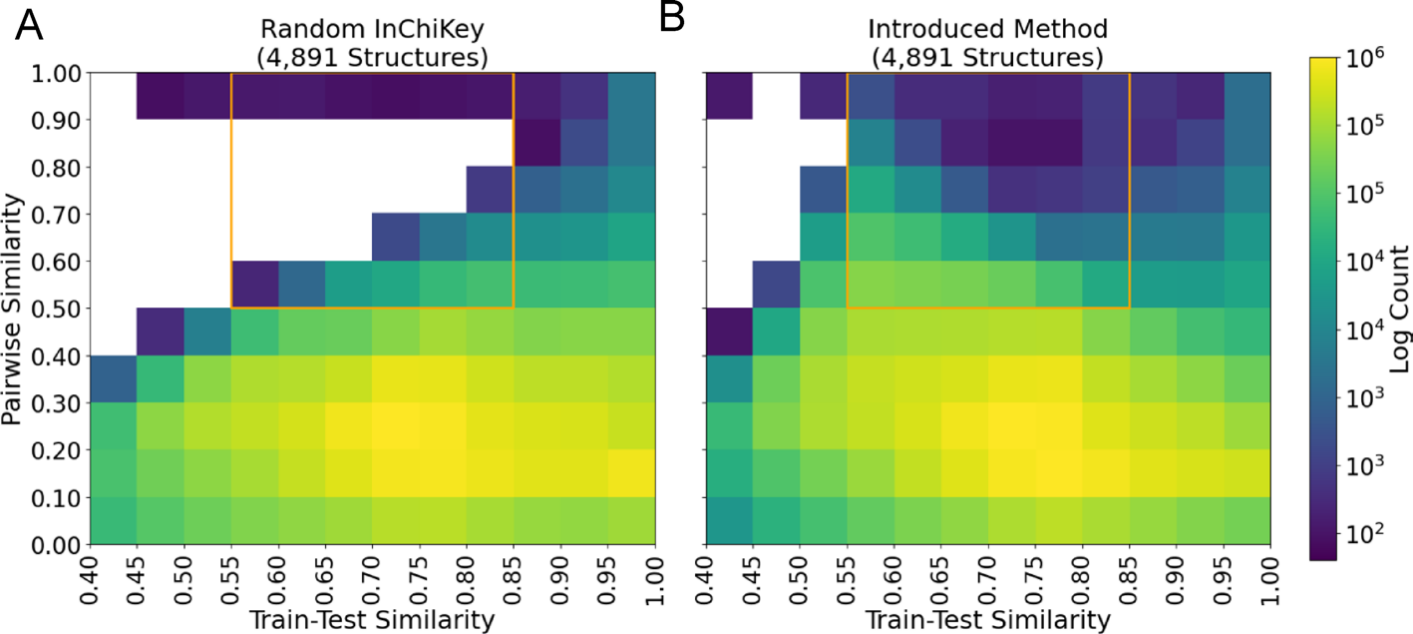
Sampling coverage. **A** The number of ordered structure pairs binned by pairwise and train-test similarity for the random sampling method. Pairwise similarity represents the Tanimoto similarity between two molecular structures. For an individual structure, train-test similarity corresponds to the maximum similarity between a test structure and all train examples. The mean train-test similarity for both structures is used to represent the train-test similarity of the pair. **B** Same as in **A** but employing our new sampling method introduced in Methods. Bins are inclusive on the left side only, with the exception of the highest bin which includes 1.0. Both test sets contain 4,734 unique InChiKeys (considering the first block only. Color represents the log base 10 count of structure pairs. Cells with counts below 200 ordered pairs (100 unique pairs) are white, highlighting poor sampling coverage. The random test set selection approach achieves a coverage of 76.7% while the introduced sampling method achieves a coverage of 92.5%. Coverage is improved in the high pairwise, moderate train-test similarity region (pairwise similarity > 0.5, train-test similarity above 0.55) denoted in orange. Unthresholded heat maps and a comparison with a 500 InChiKey random sample are available in Fig. SI 2

To build on this baseline, we introduce a method for sampling spectra and pairs designed to improve coverage of the structural similarity and train-test similarity space (Methods: Sampling Low Train-Test Similarity Pairs). This algorithm optimizes sampling of test structures over 13 train-test structural similarity bins ranging from 0.4 to 1.0 (Tanimoto similarity) by forcing structures to be placed into these bins, ensuring high coverage. This is accomplished by iteratively removing training data structures that cause test structures to have high train-test similarity. Second, to address pairwise structure similarity diversity, defined as the Tanimoto similarity between any two structures within the dataset, we apply a random walk sampling approach. This method applied to the dataset created a training set of 121,209 spectra and 17,339 structures, a validation set of 3,671 spectra and 500 structures, and a test set of 28,106 spectra and 4,891 structures. This division yielded a more complete coverage of the similar/dissimilar structure pairs and simila/dissimilar test-train distances when compared with the fully random method, even when normalizing for the number of test structures in the random method (Fig. 2). Overall, the random test set selection achieved 92 out of 120 bins while the introduced approach exceeded the threshold for 111 of 120 bins, 20.7% improvement (Fig. 2). Moreover, we define a region of interest (orange box Fig. 2) with train-test similarity (0.55, 0.85) and pairwise similarity > 0.5 that captures structurally related molecules with potential application in molecular networking, but distant from the test set. We note that the introduced method reached the threshold of 200 test pairs in all 30 bins in this region while the random selection method reached 16 out of 30 bins. For the remainder of the manuscript, we will refer to this dataset as the “All-Pairs” test dataset which contains 1,059,860,580 ordered spectrum pairs from 4,891 structures (Table 1).

**Table 1:** Dataset summary

Dataset	Structures	Spectra	Spectral pairs	Pair criteria
Train all-pairs	17,339	121,209	5,193,600*	N/A
Train filtered	16,720	106,135	5,011,200*	Ionization method, mass analyzer, adduct, Collision energy difference (if available) < 5 eV, precursor mass difference < 200 Da
Validation all-pairs	500	3671	960*	N/A
Validation filtered	480	3120	960*	Ionization method, mass analyzer, adduct, Collision energy difference (if available) < 5 eV, precursor mass difference < 200 Da
Test all-pairs	4891	32,555	1,059,860,580	N/A
Test filtered	4734	28,106	183,347,420	Ionization method, mass analyzer, adduct, Collision energy difference (if available) < 5 eV, precursor mass difference < 200 Da
Test filtered strict collision energy	1636	9199	4,511,562	Ionization method, mass analyzer, adduct, Collision energy difference < 5 eV, precursor mass difference < 200 Da

Datasets constructed in this study and used for retraining and evaluation of MS2DeepScore. Any two spectra are allowed in the All-Pairs datasets while filtered data sets require an identical ionization method, mass analyzer, and adduct. In addition, only pairs with a precursor mass difference less than 200 daltons are allowed and collision energy of spectra must be within 5 ev. For the strict collision energy filtered test set we require both spectra to have an annotated collision energy. *Training spectral pairs represent the count of pre-sampled spectrum ID pairs for training which are generated in our flexible pre-batching pipeline that produces training data conforming to a pairwise similarity target distribution. Test set pair construction is exhaustive, meaning all possible pairs of spectra are enumerated (see Methods: All-Pairs and filtered Datasets)

### MS2DeepScore evaluation

#### MS2DeepScore - reproduction

As a test case, we use the MS/MS similarity prediction method introduced with MS2DeepScore. Briefly, MS2DeepScore uses a siamese multi-layer perceptron architecture to embed two binned MS/MS spectra into two separate 200 dimensional vectors. A cosine similarity function is applied to the two embeddings to predict a structural-similarity score that approximates the Tanimoto similarity of the underlying compounds. We reproduce the training pipeline and recapitulate the relative performance here as in the original publication by retraining on the All-Pairs dataset with a test split based on random InChiKey selection (shown in Fig. SI 3, SI 2 A&C). As a consequence of the similar performance, we maintain hyperparameters consistent with the original method.

**Fig. 3:**
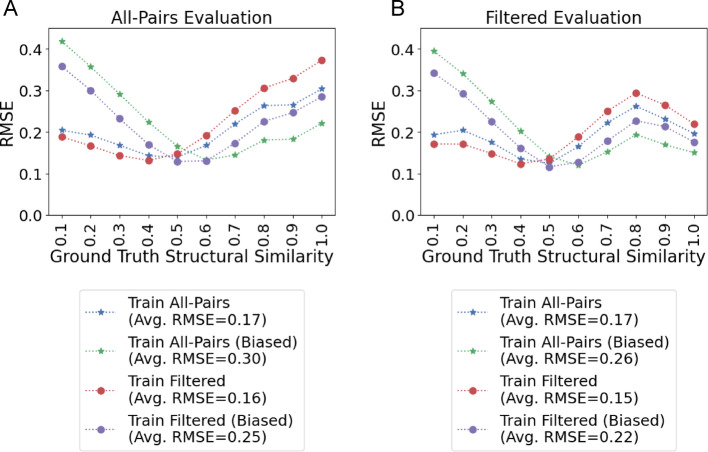
Performance relative to ground truth similarity. **A** Root mean squared error (RMSE) comparison between All-Pairs, Filtered, Unbiased, and Biased models on All-Pairs test data across 10 ground truth structural similarity bins. **B** RMSE comparison on the filtered test dataset. Across both evaluation sets, models trained on Filtered data outperform models trained on the All-Pairs set for low pairwise similarities but underperform for higher pairwise similarities. Biasing the pairwise similarity distribution during training improves pairwise similarity greater than 0.6 prediction performance. X-tick values represent the exclusive upper end of the pairwise similarity bin (e.g., 0.1 is the [0.0, 0.1) bin), with the exception of 1.0 which is inclusive

When evaluating the performance of MS2DeepScore with the All-Pairs test dataset created using the introduced methodology, we achieve an overall root mean square error (RMSE) between cosine predictions and Tanimoto scores of 0.1743 and a RMSE for molecules with pairwise structure similarity > 0.6 of 0.2630 (Fig. 3). Standard deviation is shown across 4 different random seeds in Fig. SI 4 and training loss curves are shown in SI Figure SI 5. We note that the RMSE is highest for structures with high structural similarity (Fig. 3), an observation consistent with the original publication. This is unexpected since highly similar molecular structures are expected to fragment similarly and should be easiest to predict.

### Filtered test datasets evaluation

We hypothesized that the test construction, which used all pairs of MS/MS spectra within the test set, may lead to an inflated RMSE in high similarity structures. To investigate this, we created a new “Filtered” test dataset that constrained the MS/MS pairs to having the same ionization method, mass analyzer, and adduct. Further, we constrain the collision energy difference (when available) to less than 5 eV, and the precursor mass difference to less than 200 Daltons. These constraints are reasonable because when applying MS/MS similarity in the context of tools like molecular networking, data will come from the same dataset and thus instrument conditions. This filtered set reduced the All-Pairs test set to 183,347,420 spectrum pairs from 4,734 structures (Table 1). Distributions of the number of pairs per Tanimoto score bin are shown in SI Figure SI 6.

Using this Filtered test set to evaluate the “All-Pairs model” (MS2DeepScore trained on data with no filtration criteria) performance, we find that for structural related molecules (Tanimoto similarity > 0.60), the RMSE decreases in this new evaluation (from 0.2630 to 0.2278) - (Fig. 3—Blue star line in A and B, SI Table 1). This is more striking in the highest structural similarity bin (0.9-1.0 Tanimoto similarity) where the error decreases from 0.3038 to 0.1962. The RMSE in structurally related pairs is further reduced when evaluating with a strict collision energy requirement from a bin-averaged RMSE of 0.2278 to 0.1777 although the decrease in the 0.9-1.0 bin is less significant from 0.1962 to 0.1783 (SI Note 1: Strict Collision Energy Evaluation, Fig. SI 7).

**Fig. 4:**
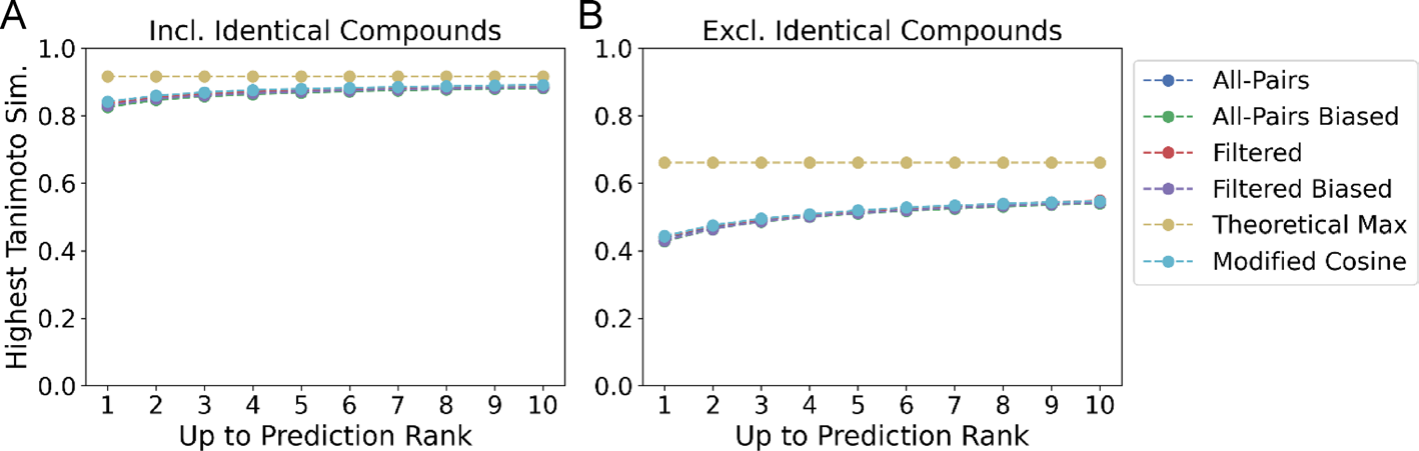
Tanimoto similarity of top predictions on filtered data. **A** The maximum Tanimoto similarity of the top k compounds with highest predicted similarity averaged across test data points. The optimal structural similarity (Theoretical Maximum) that demonstrates an upper bound on performance is also included. **B** Tanimoto similarity excluding any compounds with an identical planar 2D structure. On average, all variants of MS2DeepScore retrieve data points with comparable Tanimoto similarity to the modified cosine similarity

To provide a more realistic evaluation of performance, we introduce two new metrics that address ranking and retrieval performance that are useful in MS/MS library and analog search: *Top Candidate Similarity* and *Top-Rank*. *Top Candidate Similarity* reflects how well a model can retrieve highly similar structures (see Methods: Ranking and Retrieval Evaluation). This metric measures the maximum structural similarity of the retrieved structures to the structure corresponding to the query spectrum, up to rank k as measured by the Tanimoto Similarity. We report *Top Candidate Similarity* for the top 1, 3, and 10 candidates retrieved by each model, along with the “Theoretical Maximum,” the maximum Tanimoto similarity between all queries and distinct spectra within the test set. The “All-Pairs model” evaluated on the Filtered dataset achieves an average Tanimoto similarity of 0.8505 at k = 1, 0.8619 at k = 3, and 0.8847 at k = 10 compared to an optimal score of 0.9168 when identical structures are included in the evaluation (Fig. 4A). In the setting where no identical InChiKeys are present, the model achieves a score of 0.4703 at k = 1, 0.4909 at k = 3, and 0.5456 at k = 10, lagging behind the optimal average Tanimoto score of 0.6604 (Fig. 4B). The “All-Pairs model” performs comparably with the Modified Cosine method [10] which achieves a score of 0.8812 (vs 0.8847) and 0.5401 (vs 0.5456) at k = 10 when including and not including identical compounds respectively. Reported metrics for MS2DeepScore are averaged across four random seeds. Standard deviations are reported in SI figure SI 8, SI Table 2.

**Fig. 5:**
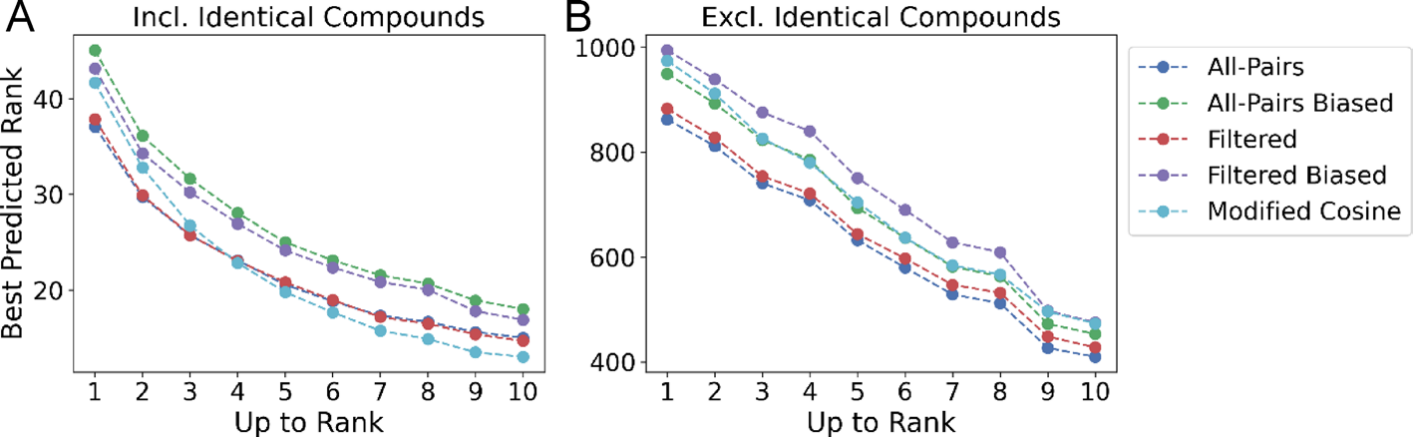
Top predicted rank on filtered data. **A** The best predicted rank for the k most similar structures, averaged across four random seeds (See: Methods: Ranking and Retrieval Evaluation). The “All-Pairs model” and “Filtered model” retrieve the highest similarity structures up to ground-truth rank k = 3, at which point they are comparable to modified cosine. **B** The best predicted rank for the k most similar structures, excluding identical structures. The “All-Pairs model” outperforms all other methods at any maximum k value

While the *Top Candidate Similarity* reflects a model’s ability to retrieve similar structures, it is limited in the ability to assess how well a model can retrieve and rank structures relative to Tanimoto similarity. To address this, we introduce the *Top-Rank* metric which measures how well a model ranks the most similar structures, within the top 1,3, and 10 candidates (see Methods: Ranking and Retrieval Evaluation). One limitation of this metric, however, is that the ranking of results corresponding to a structurally unique compound are weighted equally to those with many near-neighbors. When evaluating the “All-Pairs model” on the Filtered dataset with identical compounds included, the average rank is 37.11 at k = 1, 25.75 at k = 3, and 13.86 at k = 10 (Fig. 5A, SI Table 3). This suggests that while the model is able to retrieve identical structures, with the *Top Candidate Similarity* close to the optimal at all k, the “All-Pairs model” fails to correctly rank the absolute most similar retrieved structure. When removing identical InChiKeys from the evaluation, the best predicted rank increases to 862.56 at k = 1, 740.32 at k = 3, and 409.63 at k = 10 (Fig. 5B; Table 2, SI Table 3). In comparison to Modified Cosine, the “All-Pairs model” outerperforms with a ranking of 453.3658 to 473.26 at k = 10. Reported metrics are averaged across four random seeds. Standard deviations are shown in SI Figure SI 9. This suggests while the “All-Pairs model” can reidentify known compounds, it fails to retrieve the most similar structures.

**Table 2:** Comprehensive evaluation of the models

				Top candidate similarity (↑)		Top rank (↓)	
Training Set	Test set	Biased?	Test RMSE (↓)	k = 1	k = 10	k = 1	k = 10
All-pairs	All-pairs	N	0.1743	**0.4755**	**0.5537**	**3299.0360**	**1237.4284**
All-pairs	All-pairs	Y	0.2318	0.4663	0.5470	3975.2109	1501.2471
Filtered	All-pairs	N	**0.1584**	0.4721	0.5519	3794.1036	1381.1561
Filtered	All-pairs	Y	0.2508	0.4640	0.5462	4295.5339	1577.1777
All-pairs	Filtered	N	0.1670	0.4703	0.5456	**862.5562**	**409.6317**
All-pairs	Filtered	Y	0.2135	0.4641	0.5396	949.8339	453.3658
Filtered	Filtered	N	**0.1509**	**0.4725**	**0.5477**	882.6153	427.6419
Filtered	Filtered	Y	0.2179	0.4651	0.5418	994.7146	475.3261

The performance of the “All-Pairs” and “filtered model” trained on biased and unbiased data evaluated on the All-Pairs and filtered test sets. Reported ranking metrics exclude identical structures in the retrieval setting and arrows denote the direction of improvement for metrics. While the “unbiased filtered model” achieves the best All-Pairs test RMSE, *Top candidate similarity* only marginally increases in comparison to the “biased filtered model.” however, on the *Top rank* metric, the “unbiased All-Pairs model” outperforms all others. Similarly, on the filtered test set, the “filtered model” again achieves the best test RMSE with limited change in *Top candidate similarity*. Again, the “unbiased All-Pairs model” performs best on the *Top rank* evaluation for the filtered test set. Bold numbers indicate highest performance within each test set.

### Filtered training datasets evaluation

**Fig. 6:**
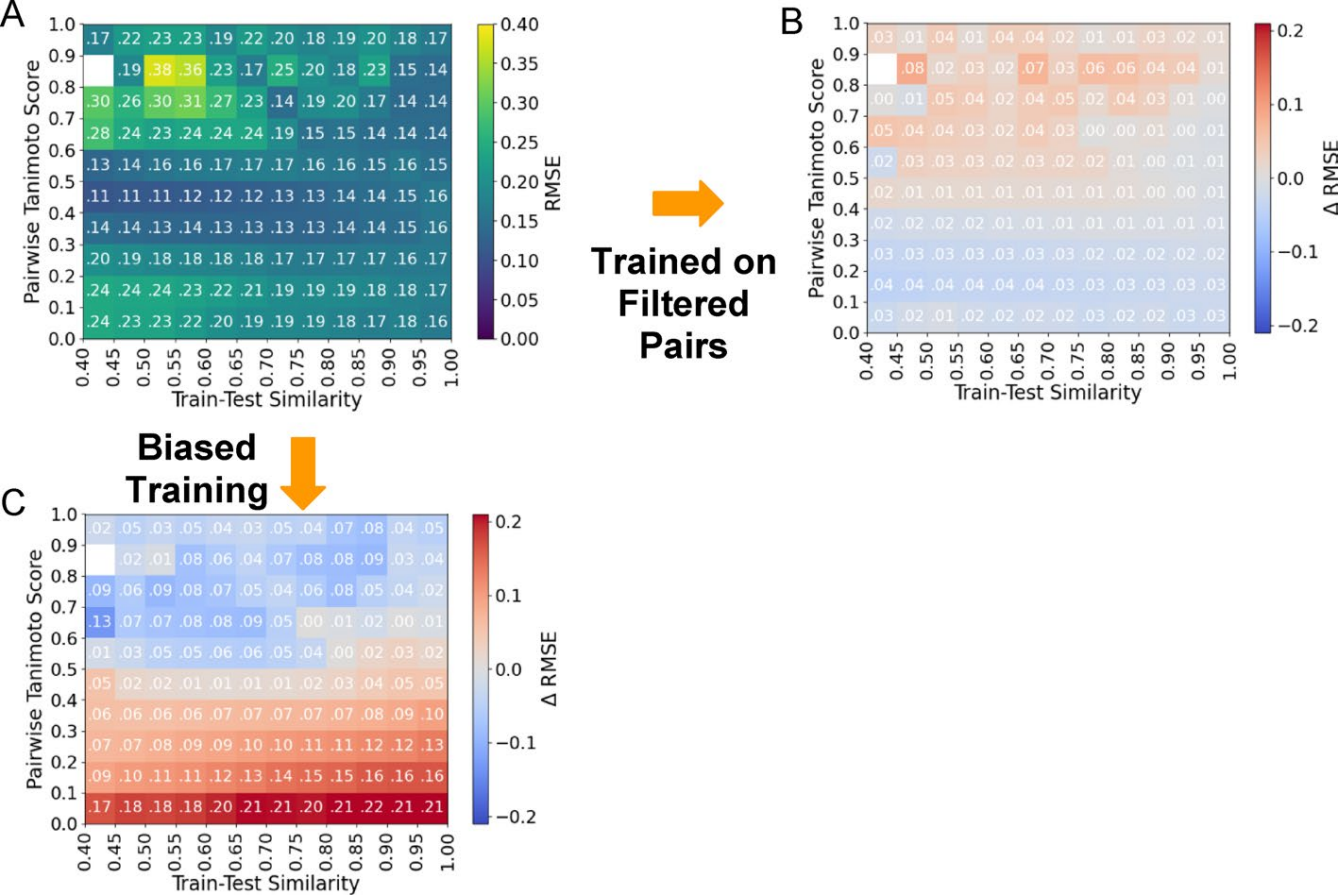
Effect of training methodology refinements. **A** The RMSE of the “All-Pairs Model” evaluated on the Filtered dataset. **B** Training with filtered MS/MS pairs results in a decrease in average RMSE of 0.0161. However, this decrease was not uniformly beneficial to all pairs of spectra in the test data; RMSE increased for in areas with pairwise similarity higher than 0.6 and decreased for low pairwise similarity (< 0.3). **C** The change in RMSE for the “Biased All-Pairs model” in comparison to the unbiased “All-Pairs model” shown in (**A**). Biased training improves RMSE for high pairwise similarity pairs (> 0.6) and increases RMSE for low pairwise similarity pairs (< 0.4)

With the same methodology we constructed a training set of filtered pairs. This training set reduces the All-Pairs training set from 121,209 to 106,135 spectra and 17,339 to 16,720 structures. We retrained MS2Deepscore and evaluated the performance across the Filtered evaluation set. While this new training improved the average RMSE (from 0.1908 to 0.1509, SI Table 1), in Fig. 3B, we note that the bin-averaged RMSE in the structurally related molecules (Tanimoto score > 0.6) increases significantly from 0.2278 to 0.2569. Notably, the effect of training on filtered data on RMSE remains consistent across train-test similarities (Fig. 6). From a practical perspective, the higher structural similarity ranges are more important for downstream matching applications, e.g. MS/MS library search and molecular networking. Thus, even though the average RMSE improvement is notable, in this scenario, it is likely less useful of a metric to understand how these similarity models may be useful in practice (for further discussion see SI Note 2). We argue that focusing on higher-structural similarity diversity bins would be of more practical value, in the > 0.6 or > 0.7 Tanimoto ranges.

### Biased training increases prediction accuracy in highly structurally similar molecules

Despite improved overall RMSE, training on Filtered data failed to improve performance on structurally related molecules. Therefore, we bias the training distribution for the “All-Pairs model” towards higher pairwise similarity pairs to improve performance on such pairs (See Methods: Biased Training of MS2DeepScore). Our target distribution for training data contains 50% of pairs with a pairwise similarity > 0.81 Tanimoto score while the random sampling scheme introduced in (Huber et al. 2021) targets 19%. However, we replicate the MS2DeepScore sampling methodology which first prioritizes complete coverage of InChiKeys and secondarily prioritizes the distribution of pairwise similarities at training time (Fig. SI 10a). During the selection of the All-Pairs training data targeting a uniform distribution, 9.75% of training samples are above the 0.81 Tanimoto similarity threshold while during the biased sampling, 24.57% of training pairs are above the threshold (Fig. SI 10).

For the “All-Pairs model” evaluated on the Filtered test set, biasing results in an increased overall RMSE of 0.2587 from 0.1670. However, this retraining results in a decrease in bin-averaged RMSE for structurally related molecules (ground truth similarity > 0.6) from 0.2630 to 0.1823 (Fig. 3A) when training and evaluating on the All-Pairs dataset and 0.2278 to 0.1663 when training on All-Pairs and evaluating on the Filtered dataset (Fig. 3B). Further, we observe that the reduction in error for high structural similarity pairs is maintained across train-test similarity (Fig. 6). Notably, the 0.9-1.0 bin of the “All-Pairs model” evaluated on the Filtered data set achieves the largest decrease in RMSE of 0.0461.

Despite the improvement in RMSE for structurally related compounds, there was no improvement in the retrieval setting when biasing the training data. For the “All-Pairs model,” biased training data marginally improved *Top Candidate Similarity* when including identical structures from 0.8505 to 0.8464 at k = 1 and from 0.8847 to 0.8816 at k = 10 (Fig. 4A, SI Table 2). A similar pattern holds when identical structures were excluded from the retrieval metrics in which case the “Biased All-Pairs model” marginally improved (Fig. 4B; Table 2), achieving a score of 0.5396 versus 0.5456 for the “Unbiased All-Pairs model” at k = 10.

Similarly, *Top Rank* performance decreased, with the average rank increasing from 13.86 to 18.02 at k = 10 (Fig. 5A, SI Table 3) when spectra from identical InChiKeys were present in the retrieval. In addition, “Biased All-Pairs model” performance was consistently worse than the Modified Cosine score (Fig. 5A, SI Table 3), achieving a rank of 18.02 versus 13.0057 at k = 10. When excluding identical compounds, the performance for the “All-Pairs model” trained on biased data was comparable to Modified Cosine, 453.37 versus 473.26 at k = 10, but performed worse than the “Unbiased All-Pairs model” which achieved a score of 409.63 at k = 10 (Table 2. SI Table 3).

### Generalization of MS2DeepScore

Enabled by the improved sampling for higher pairwise similarity structures distant from the train set, we probe the generalization capabilities of the “All-Pairs model” on the All-Pairs test set (Fig. SI 11a) and the Filtered test set (Fig. SI 11b). On the All-Pairs test set, RMSE increased from 0.16 to 0.30 for the [0.7, 0.8) pairwise similarity bin, and similarly from 0.18 to 0.23 for the [0, 0.1) bin. Meanwhile, in the [0.3, 0.5) pairwise similarity region, there is limited change in RMSE as train-test similarity changes with RMSE decreasing from 0.16 to 0.11 in the [0.4,0.5) range (Fig. SI 11a). Similarly, when evaluating on the Filtered dataset, RMSE increased from 0.14 to 0.31 for the [0.7,0.8) pairwise similarity bin and 0.16 to 0.24 for the [0, 0.1) bin while decreasing from 0.16 to 0.11 for the [0.4, 0.5) range (Fig. SI 11b). This suggests that not only is the performance of the model out of distribution affected by the train-test similarity, but also the pairwise structural similarity of the input data. A comparison with the “Filtered model” is included in SI Figure SI 12.

**Fig. 7:**
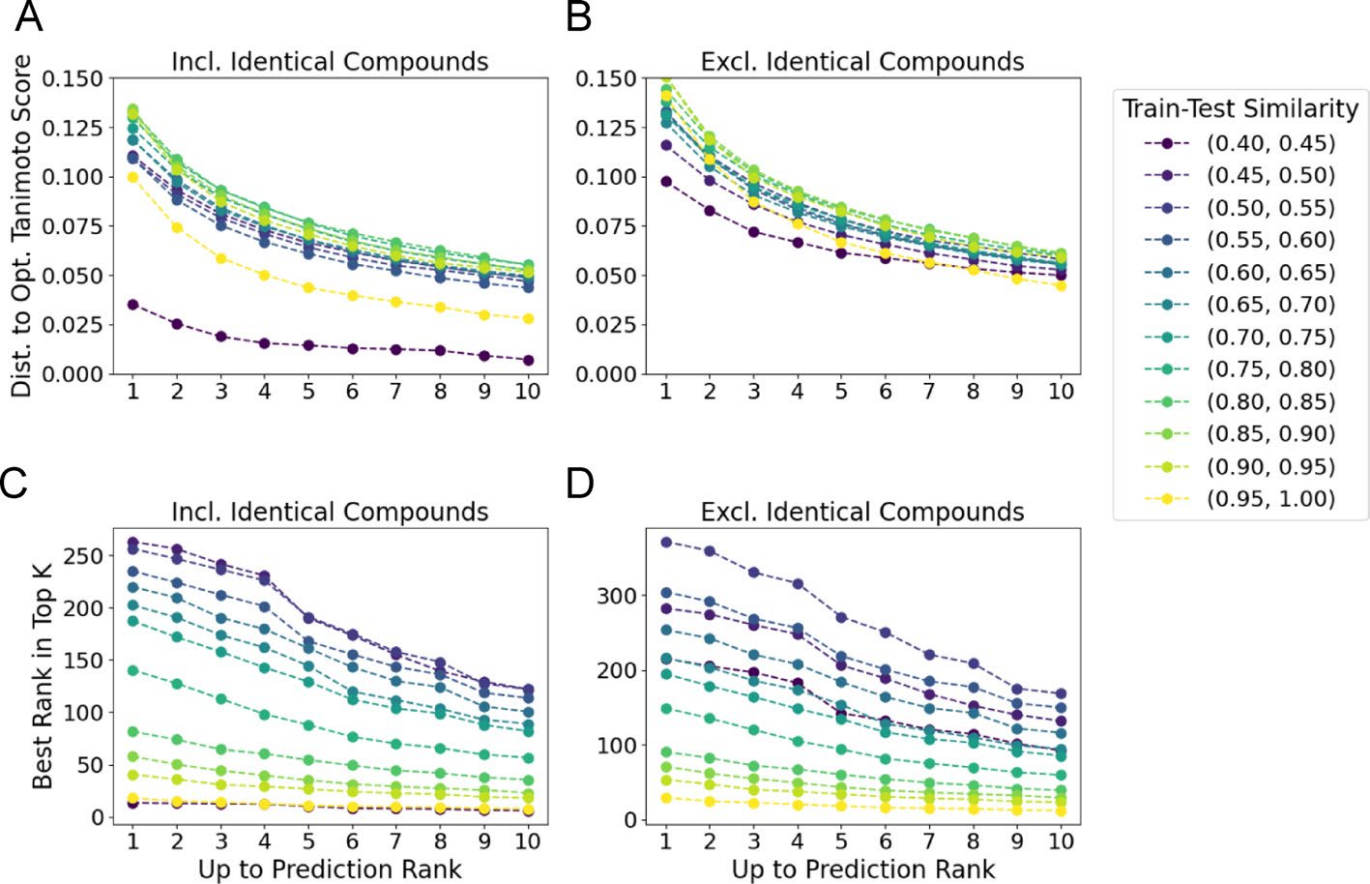
Ranking and retrieval metrics by train-test similarity. **A** & **B** Distance between the maximum predicted Tanimoto similarity up to rank k and the optimal Tanimoto similarity plotted for each train-test similarity bin. **A** When including identical compounds, the most similar bin (0.95, 1.0) outperforms all other bins (with lower bounds greater than 0.55) where the 200 pair sampling threshold was achieved (Fig. 2). **B** When excluding identical compounds, the same trend holds, although when considering the top 10 predictions, the closest bin to the test set (0.95, 1.0) outperforms all others. C&D) The best predicted rank for the top-k most similar structures to each query when identical compounds are included in the results (**C**), and when excluded (**D**). In both cases, the model predicts better (lower) ranks for the most similar compounds as the data points become closer to the training set

Further, we investigate the role of train-test similarity in ranking and retrieval on the Filtered evaluation set using the “All-Pairs model”. For the *Top Candidate Similarity* metric, we report distance to the optimal structure similarity for each train-test similarity bin in (Fig. 7A, B). In the case where identical structures are included in the retrieval set (Fig. 7A), the most similar train-test bin (0.95, 1.0 Tanimoto) measured the smallest gap to the optimal structural similarity (Fig. 7A). When excluding identical structures from retrieval (Fig. 7B), we still observe that the top train-test similarity bin (0.95,1.0) exhibits the smallest gap to the optimal similarity. However, outside of this top window, we do not observe a clear trend for the other bins with a train-test similarity less than 0.95.

In contrast, for the *Top Rank* metric, there is a decrease in performance as train-test similarity decreases. When including identical compounds in retrieval, at k = 1, for the most similar train-test bin, the “All-Pairs model” predicts an average rank of 17.95, while the (0.55,0.60) bin predicts an average rank of 234.97 (Fig. 7C). This trend holds across all k-values, with an average rank of 7.60 for (0.95, 1.0) similarity bin and 113.62 for the (0.55,0.60) bin at k = 10. When not considering identical compounds in retrieval at k = 1 the (0.95, 1.0) similarity bin has an average rank of 29.30 while the (0.55,0.60) has an average rank of 304.55 (Fig. 7D). For k = 10, the “All-Pairs model” predicts an average rank of 11.9680 and 149.77 for the (0.95, 1.0) and (0.55, 0.60) bins respectively, indicating that the “All-Pairs model” is better able to rank highly similar compounds which are closer to the training set.

## Discussion

By using an experimental condition-conscious approach to evaluation, we reduce the error for structurally related compounds and explain the anomalous trend of high error for high similarity structures (those with Tanimoto similarity greater than 0.9). Further, we use a new training method that biases towards high-pairwise similarity structures that further reduces the prediction error. However, we also observe that while we enhance the prediction accuracy (measured by RMSE), other metrics measuring retrieval accuracy notably suffer. This finding suggests that an ensemble approach may improve overall performance by allowing specialized models to focus on different regions of the input space [17, 18] We hope the complementary nature of the three metrics we include in this manuscript will provide a more comprehensive evaluation of future models.

While the introduced sampling method increases the coverage of the space greater than 0.55 train-test similarity (Fig. 2, Fig. SI 2), the entirety of the two-dimensional space is still not covered. Notably, for the region of higher pairwise similarity and low training similarity (pairwise similarity > 0.5, train-test similarity < 0.55), the introduced test-train selection method fails to meet the 200 pair criteria. This region is especially challenging as the test data is very dissimilar from the training data, which artificially forces the training dataset to be smaller. Thus, we have chosen a compromise to balance this region with an acceptable training dataset size. Since we are releasing an open-source pipeline that is continually rerun as new libraries are released, we anticipate that additional and new libraries will help increase this coverage while maintaining large training datasets.

Further we would like to emphasize that molecular similarity is a subjective measure with thresholds varying by application, [19–21] for this reason we offer a benchmark across train-test similarities and pairwise similarities, enabling end users to make an informed decision on the necessity for retraining for their specific task. In a similar vein, different levels of error may be appropriate for different tasks and a particular use case should inform users on the necessity to retrain their models. For example, while the structural similarity of retrieved compounds is roughly independent of train-test similarity, the ranking of retrieved compounds suffers (Fig. 7A, C).

One weakness of the community data included here is the incompleteness of collision energies. While we used the original provenance files for the public library creation to collect as many collision energies as possible, 70.72% of the test spectra do not have collision energy metadata. As a compromise, we allow pairs without collision energy information to maximize the size of the training and test datasets but note that requiring all pairs to have annotated collision energies can reduce error in high-pairwise similarity structures (Filtered Evaluation, SI: Strict Collision Energy Evaluation).

## Conclusions

We present a publicly available training and test dataset al.ong with evaluation techniques and metrics that target performance characteristics meaningful to the practical applications of MS/MS structural similarity. Further, we experiment with two domain-inspired training methodologies and demonstrate orthogonality in the proposed metrics. We anticipate that our benchmark will support the rapid development of new models and enable their comprehensive assessment. Finally, we hope that this work and dataset can complement existing efforts [22] in the field to create standardized datasets and machine learning problem formulations.

## Methods

### Dataset processing & cleaning

To generate the dataset, we collect data from the GNPS Libraries [10] and MassBank EU [11]. Each spectrum, stored in JSON and MGF format, is associated with a metadata CSV table by a unique identifier from their respective databases. For GNPS spectra, we reparse the provenance files in order to collect additional metadata associated with each of the spectra. Additionally, many GNPS library entries contain collision energies as a part of the compound name or instrument field, for each of these the collision energy is parsed out by a manually derived set of rules (e.g., compound names in the BERKELEY-LAB library contain strings such as “CollisionEnergy:35”). Adduct annotations are harmonized via a manually curated list available in the GitHub repository in which we prioritize the most frequent adducts in the data. Internally, the pipeline passes spectra in an MGF format and metadata as a CSV file. Structures are stored as both InChiKeys and SMILES strings. The final output of the pipeline is available as a JSON file, or alternatively a CSV and MGF file representing metadata and spectra respectively.

To standardize the 2D structures we did the following. First, we removed salts in the 2D structure by choosing the largest covalently bonded fragment using RDKit [23]. Second, we neutralized the molecules to ensure the 2D structure represents a de-charged representation (e.g. M, instead of M + H for M + H adducts). Third, we remove stereochemistry. Finally, we resolve ambiguity of structures arising from multiple tautomers [24] by enumerating all potential tautomers and choosing the most energetically stable using RDKit. We validate annotations by comparing the calculated monoisotopic mass of the annotations with their adducts against the experimental precursor masses in the spectra, removing any entries with an error between annotation and precursor m/z greater than 50 ppm.

Finally, instrument annotations available from GNPS are propagated to ionization, mass analyzer, dissociation method, and manufacturer columns via a manually defined set of substring criteria and propagation rules. For example, an annotation of “CID, ESI, Q-Exactive” would be assigned the dissociation method of CID, the ionization method of ESI, a mass analyzer value of Orbitrap, and a manufacturer annotation of Thermo. When metadata for ionization, mass analyzer, and dissociation method was available in the provenance files, this value was taken over the user annotations.

### Selecting MS/MS data for machine learning

We discarded the GNPS user uploaded libraries and the BMDMS GNPS import. Following (Huber et al. 2021) all entries without structures and precursor masses greater than 1500 Daltons were discarded. We preprocessed spectra by rescaling intensities, removing peaks below 0.1% relative intensity and outside the range of 10–2000 m/z. Finally, we select spectra with “significant fragmentation”, those with greater than 4 peaks with a relative intensity greater than 2%.

### Sampling low train-test similarity pairs

We generate an evaluation set with a focus on low train-test similarity structures. Distance between molecular structures is calculated as the Tanimoto similarity between 2048 bit RDKit Daylight fingerprints [25]. Train-test similarity for each structure is the maximum pairwise similarity between that test structure and all train structures. The goal of the algorithm is to optimize the number of highly distant test structures sampled. For the purposes of test set selection, we use 13 bins from [0.4, 1] for train-test similarity. The method is as follows:

1. *Selecting low train-test similarity structures* In order to select spectra from pairs of structures highly dissimilar from the training set, we select individual structures that meet this criterion. *Alg*: Calculate the all-pair similarity matrix between unique structures. Create a histogram, binning each structure by the maximum similarity between it and any other data point. Randomly select the first *data_points_per_bin* from each of the histogram bins as the starting point to fill the 13 train-test similarity bins.
2. *Selecting high-pairwise similarity structures* In order to ensure sufficient coverage of high pairwise similarity structures, we introduce an additional selection step. *Alg*: Initialize a graph in which nodes represent all structures in the dataset and edges represent ground-truth structural similarity greater than 0.7. Randomly select *num_test_roots* with degree greater than three. From each root, perform a random walk of length 4 moving all roots and structures visited during the random walk to the test set.
3. *Balancing low train-test similarity structures* Combine the unique set of selected structures from steps one and two as the initial test set. To enforce low train-test similarity, we iteratively remove samples from the training set to reduce similarity between train and test data points. We bin the test structures into 13 evenly spaced train-test similarity bins between 0.4 and 1.0. Beginning with the first underfilled bin, sample structures in the next non-empty bin as the data points to move. For each data point to move, remove all structures from the training set with a similarity to the data point being moved greater than the upper bound in the current bin. For example, consider the [0.5, 0.55) bin which is underfilled by three structures. Three structures will be sampled from the [0.55, 0.6) bin and all training structures with greater than 0.55 similarity to those structures will be removed, shifting these data points to the [0.5,0.55) bin. This process is repeated until all bins have reached *data_points_per_bin* or until 80% of the training set is eroded.

The number of spectra taken in the first and second phases of the algorithm were empirically chosen to achieve 80% coverage of the pairwise and train-test grid shown in Fig. 1. Similarities for pairs of test spectra to the training set is quantified as the average train-test similarity of the two corresponding structures.

### All-Pairs and filtered datasets

To explore the role that instrumentation and experimental parameters play in elevating error in MS/MS similarity prediction, we introduce two datasets. The first dataset, “All-Pairs” allows for any pair of spectra to be included in the training and evaluation datasets. This setting is consistent with the original training of MS2DeepScore. The second dataset, “Filtered”, requires pairs of spectra in the training and evaluation sets to be generated from instruments with the same mass analyzer and ionization method. Further, it requires the collision energy to be within 5 eV, although due to the sparsity of collision energy labels, if collision energy is missing for either spectrum, we remove this criterion. Finally, we remove pairs with a precursor mass difference greater than 200 Daltons and require pairs to have identical adducts. For training datasets, the net result is an HDF file in which each key corresponds to a table containing one epoch of training data. The table is composed of a pair of spectra ids used to reference MS/MS data along with a pair of InChiKeys and the target similarity. Training data is produced by a Nextflow [26] workflow allowing for reproducible and highly-scalable pre-batching of data. Pairs of test structures are saved as parquet files containing InChiKeys, spectrum ids, target similarity scores, and train-test similarity for each pair.

### Ranking and retrieval evaluation

To provide a more comprehensive evaluation of models, we simulate a database retrieval scenario within our test set. To this end, we propose two new metrics to evaluate our model: (1) Top Candidate Similarity (Fig. 4) and (2) Top-Rank (Fig. 5), averaging results over four random seeds. As a baseline, we compute these metrics using the MatchMS [27] implementation of Modified Cosine with a fragment tolerance [28] of 0.10 an m/z power of 0.0, and an intensity power of 0.50.

#### Top candidate similarity

To assess whether a method is capable of retrieving highly similar structures we report the maximum structural similarity between all predictions up to each rank k and their query spectra. One limitation, however, is that spectra corresponding to structures highly dissimilar from all others in the test set will skew the average structural similarities downwards. Therefore, we report the average of the kth highest similarities over all query structures in the dataset as the “theoretical maximum.” To evaluate the model’s performance in both a re-identification and analogue search setting we evaluate the model on both the full dataset, considering all pairs allowed by the filtering criteria, and a reduced dataset in which we remove all pairs with identical InChiKeys.

#### Top-rank

To assess the performance of the methods in an application-oriented setting agnostic to the true similarity distribution, we propose an additional metric that reflects the ability to correctly rank the most similar structure in the top-k predictions for each query. In this metric, we take the minimum predicted rank for the top-k most similar structures, averaging it across all queries. As an example, if the top three most similar structures for a given query have a predicted ranking of [21, 42, 13], the metric reports 13. Ties are grouped as a set, rather than ranking independently. Concretely, for a given query, if there are multiple InChiKeys that correspond to the same ground truth similarity value, we assign all of those InChiKeys to rank j and assign the next most similar keys to rank j + 1.

### MS2DeepScore training

Consistent with the original manuscript, we train an MS2DeepScore model using an Adam optimizer (lr = 0.001, batch size = 32) for 150 epochs using early stopping (patience = 10 epochs) to mitigate overfitting. On our All-Pairs test dataset, we achieve an average root mean squared error (RMSE) of 0.1785. The trends in error dependent on ground truth similarity are in agreement with the original manuscript (Fig. 2A).

### Biased training of MS2DeepScore

While broadly predicting structural similarity serves as a reasonable training target for machine learning models, accuracy on high ground-truth similarity pairs is the most important in practical applications. Widely used methods such as molecular networking [2] and analog search [1] rely on the inference of highly similar structures and stand to gain the most from improved structural similarity prediction methods. Therefore, we explore the role of biasing the pairwise similarity distribution during training as a means for improving error on high similarity pairs.

During standard training, pairwise similarities are binned into ten equally spaced bins and bin indices are randomly selected to produce a roughly uniform distribution over pairwise similarities. Instead, we implement unequal width bins to bias the sampling towards higher pairwise similarities by applying an exponent of 0.3 to the bin edges. In practice, this means 50% of pairs seen during training have a ground truth similarity greater than 0.81. However, due to the highly right-skew pairwise similarity distribution, broader bins in the lower end of the distribution fail to proportionally sample the upper end of their range. To mitigate this effect, we use 20 bins such that the sampled distribution closer approximates the target distribution although the artifact of this approach is still visible in the lowest bin.

## Electronic supplementary material

Below is the link to the electronic supplementary material.

Supplementary Material 1

## Data Availability

Source code for the data harmonization portion of the pipeline is available at https://github.com/Wang-Bioinformatics-Lab/gnps_ml_processing_workflow. Source code for benchmarking is available at https://github.com/Wang-Bioinformatics-Lab/Baselines_For_Benchmark. Harmonized data is updated dailyand is publicly available at https://external.gnps2.org/gnpslibrary. The selected data (including a full list of GNPS and MassBank identifiers) along with the training and test sets used for these experiments is published as a Zenodo record at https://zenodo.org/records/15522127The GNPS and MassBank logos are licensed under the Creative Commons Attribution 4.0 International License (CC BY 4.0) and can be attained from https://ccms-ucsd.github.io/GNPSDocumentation/logos/ and https://commons.wikimedia.org/wiki/File: MassBank_logo.png respectively.Source data from GNPS can be found at https://gnps.ucsd.edu/ProteoSAFe/libraries.jspSource data from MassBank can be found at https://massbank.eu/MassBank/.

## References

[1] Watrous J, et al. Mass spectral molecular networking of living microbial colonies. Proc Natl Acad Sci. 2012;109. 10.1073/pnas.1203689109 PMC3387089 22586093

[2] Nothias L-F, et al. Feature-based molecular networking in the GNPS analysis environment. Nat Methods. 2020;17:905–8. 32839597 10.1038/s41592-020-0933-6 PMC7885687

[3] Stein SE, Scott DR. Optimization and testing of mass spectral library search algorithms for compound identification. J Am Soc Mass Spectrom. 1994;5:859–66. 24222034 10.1016/1044-0305(94)87009-8

[4] Li Y, et al. Spectral entropy outperforms MS/MS Dot product similarity for small-molecule compound identification. Nat Methods. 2021;18:1524–31. 34857935 10.1038/s41592-021-01331-z PMC11492813

[5] Wang X, et al. Network topology evaluation and transitive alignments for molecular networking. J Am Soc Mass Spectrom. 2024;35:2165–75. 39133821 10.1021/jasms.4c00208 PMC11516331

[6] Bushuiev R, et al. Self-supervised learning of molecular representations from millions of tandem mass spectra using DreaMS. Nat Biotechnol. 2025; 10.1038/s41587-025-02663-3 10.1038/s41587-025-02663-3 PMC13090125 40410407

[7] Huber F et al. Spec2Vec: improved mass spectral similarity scoring through learning of structural relationships. PLoS Comput Biol. 2021;17. 10.1371/journal.pcbi.1008724 PMC7909622 33591968

[8] Huber F, van der Burg S, van der Hooft JJJ, Ridder L. MS2DeepScore: a novel deep learning similarity measure to compare tandem mass spectra. J Cheminform. 2021;13. 10.1186/s13321-021-00558-4 PMC8556919 34715914

[9] Guo H, Xue K, Sun H, Jiang W, Pu S. Contrastive learning-based embedder for the representation of tandem mass spectra. Anal Chem. 2023;95:7888–96. 37172113 10.1021/acs.analchem.3c00260

[10] Wang M, et al. Sharing and community curation of mass spectrometry data with global natural products social molecular networking. Nat Biotechnol. 2016;34:828–37. 27504778 10.1038/nbt.3597 PMC5321674

[11] Horai H, et al. MassBank: a public repository for sharing mass spectral data for life sciences. J Mass Spectrom. 2010;45:703–14. 20623627 10.1002/jms.1777

[12] Ye N et al. OoD-Bench: quantifying and Understanding two dimensions of Out-of-Distribution generalization. 2021. Preprint at 10.48550/ARXIV.2106.03721.

[13] Yang J, Zhou K, Li Y, Liu Z. Generalized out-of-distribution detection: a survey. Int J Comput Vis. 2024;132:5635–62.

[14] Zhao B, et al. OOD-CV-v2: an extended benchmark for robustness to Out-of-Distribution shifts of individual nuisances in natural images. IEEE Trans Pattern Anal Mach Intell. 2024;46:11104–18. 39288047 10.1109/TPAMI.2024.3462293

[15] Hupkes D, et al. A taxonomy and review of generalization research in NLP. Nat Mach Intell. 2023;5:1161–74.

[16] De Jonge NF, et al. Reproducible MS/MS library cleaning pipeline in matchms. J Cheminform. 2024;16:88. 10.1186/s13321-024-00878-1 PMC11285329 39075613

[17] Jacobs RA, Jordan MI, Nowlan SJ, Hinton GE. Adaptive mixtures of local experts. Neural Comput. 1991;3:79–87. 31141872 10.1162/neco.1991.3.1.79

[18] Yuksel SE, Wilson JN, Gader PD. Twenty years of mixture of experts. IEEE Trans Neural Netw Learn Syst. 2012;23:1177–93. 24807516 10.1109/TNNLS.2012.2200299

[19] López-Pérez K, et al. Molecular similarity: theory, applications, and perspectives. Artif Intell Chem. 2024;2:100077. 40124654 10.1016/j.aichem.2024.100077 PMC11928018

[20] Medina-Franco JL, Sánchez-Cruz N, López-López E, Díaz-Eufracio, BI. Progress on open chemoinformatic tools for expanding and exploring the chemical space. J Comput Aided Mol Des. 2022;36:341–54. 10.1007/s10822-021-00399-1 PMC8211976 34143323

[21] Maggiora G, Vogt M, Stumpfe D, Bajorath J. Molecular similarity in medicinal chemistry: miniperspective. J Med Chem. 2014;57:3186–204. 24151987 10.1021/jm401411z

[22] Bushuiev R, et al. MassSpecGym: A benchmark for the discovery and identification of molecules. In: Globerson A, Mackey L, Belgrave D, Fan A, Paquet U, Tomczak J, et al., editors. Advances in Neural Information Processing Systems. Curran Associates, Inc.; 2024. p. 110010–27. https://proceedings.neurips.cc/paper_files/paper/2024/file/c6c31413d5c53b7d1c343c1498734b0f-Paper-Datasets_and_Benchmarks_Track.pdf

[23] Greg, Landrum et al. rdkit/rdkit: 2024_09_1 (Q3 2024) Release. Zenodo 10.5281/ZENODO.591637 (2024).

[24] Martin YC. Let’s not forget tautomers. J Comput Aided Mol Des. 2009;23:693. 19842045 10.1007/s10822-009-9303-2 PMC2776169

[25] Bajusz D, Rácz A, Héberger K. Why is Tanimoto index an appropriate choice for fingerprint-based similarity calculations? J Cheminform. 2015;7:20. 26052348 10.1186/s13321-015-0069-3 PMC4456712

[26] Di Tommaso P, et al. Nextflow enables reproducible computational workflows. Nat Biotechnol. 2017;35:316–9. 28398311 10.1038/nbt.3820

[27] Huber F, et al. matchms - processing and similarity evaluation of mass spectrometry data. J Open Source Softw. 2020;5:2411.

[28] Bittremieux W, et al. Comparison of cosine, modified cosine, and neutral loss based spectrum alignment for discovery of structurally related molecules. J Am Soc Mass Spectrom. 2022;33:1733–44. 35960544 10.1021/jasms.2c00153

