# Supplementary material for: An evaluation methodology for machine learning-based tandem mass spectra similarity prediction
Source: BMC Bioinformatics. 2025 Jul 11;26:174. doi: 10.1186/s12859-025-06194-1 (PMC12247221; doi:10.1186/s12859-025-06194-1)
Supplement: Supplementary file 1 — Supplementary Material 1 [file 12859_2025_6194_MOESM1_ESM.docx]

**
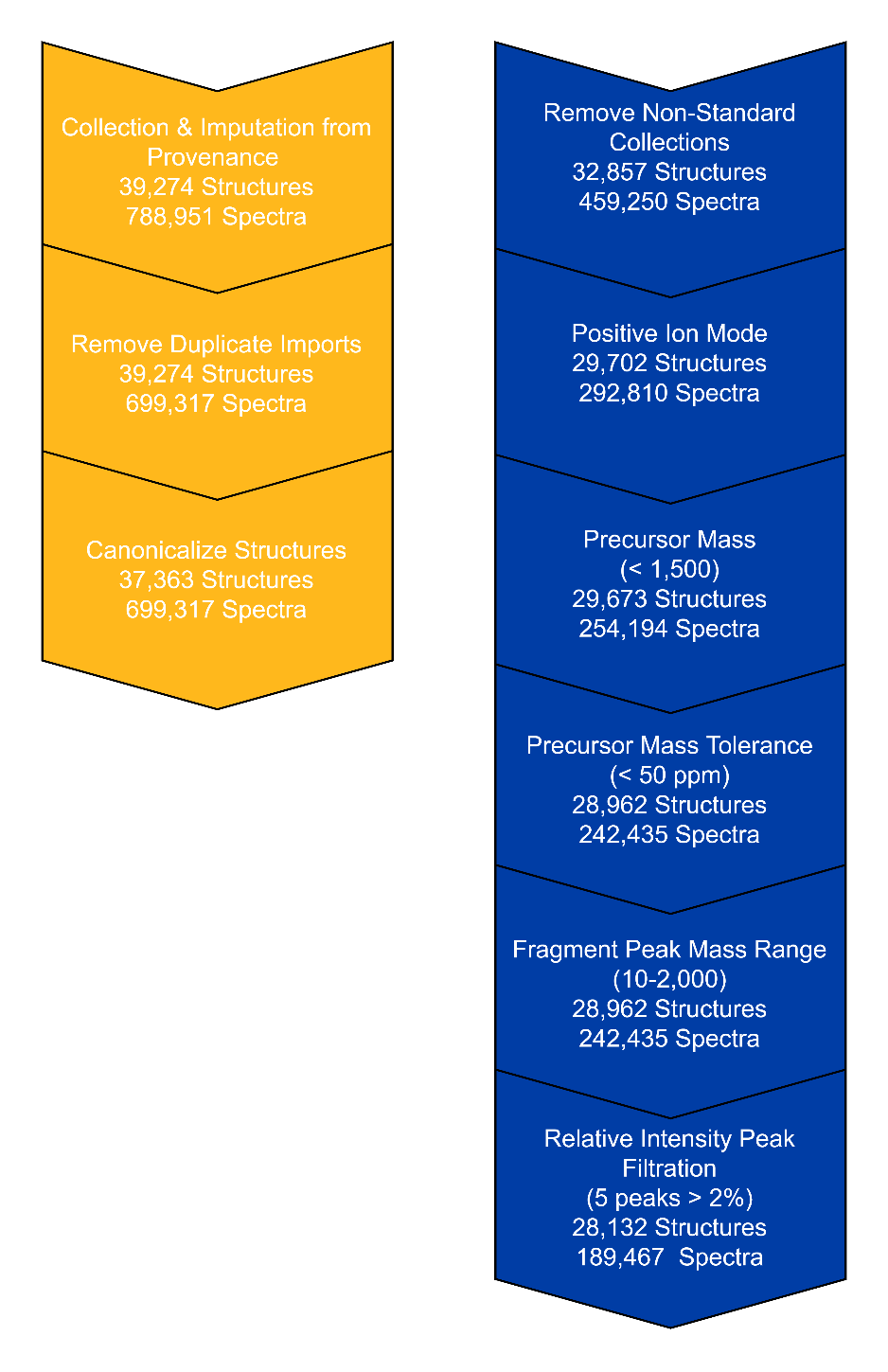
Supplementary Information**

**SI Figure 1 – Data Preparation Pipeline** A schematic of the full data described in **Methods** and **Results:** **Training/Test Data Preparation for Structural Similarity Prediction**. Portions in orange denote the pipeline described in **Methods: Dataset Processing & Cleaning** while portions in blue denote steps described in **Methods: Selecting MS/MS Data for Machine Learning**.


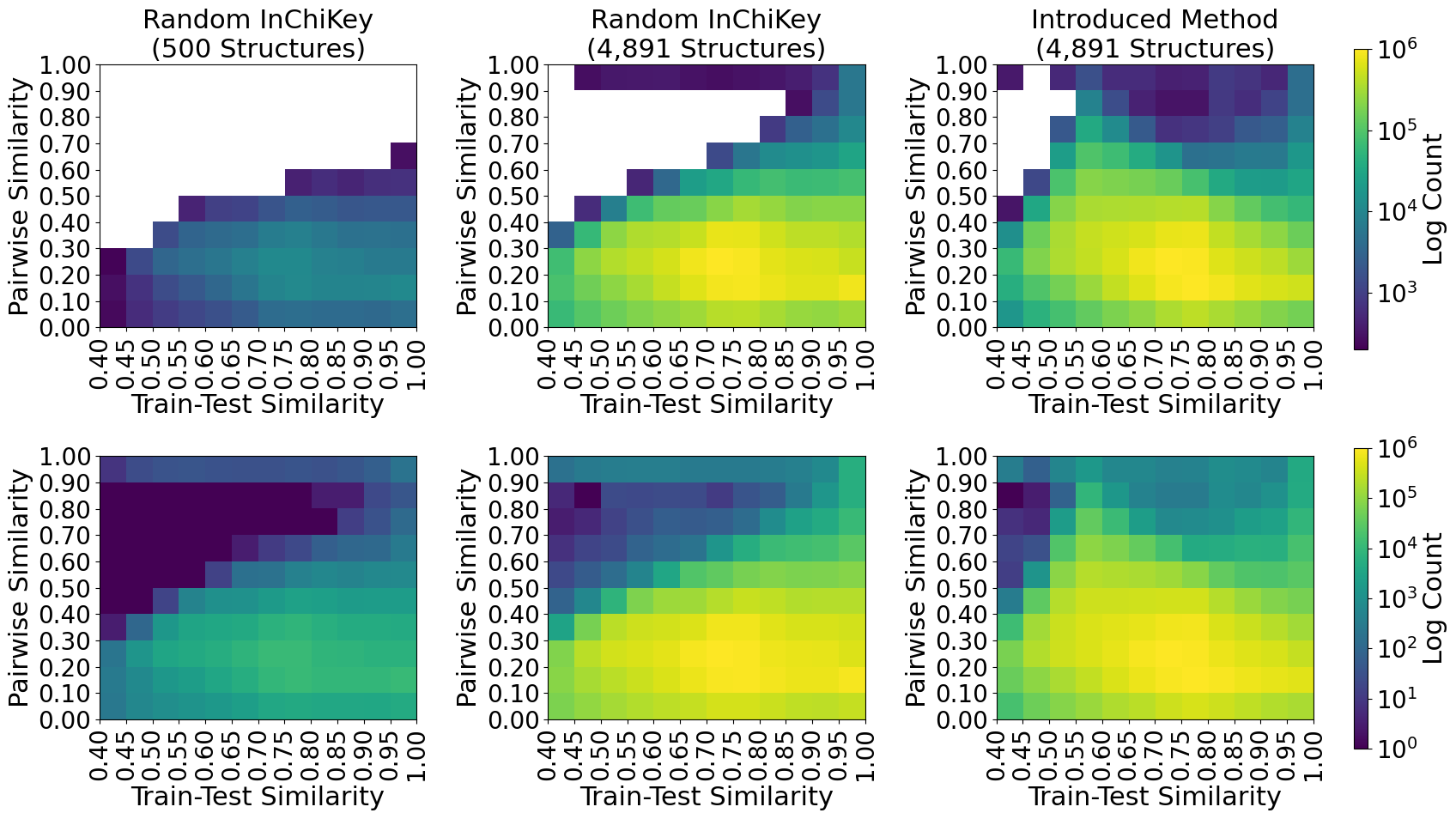


A

B

C

F

E

D

**SI Figure 2 – Comparison of Random and Introduced Sampling Method** A&B) The number of ordered pairs of structures binned by pairwise and train-test similarity for the random sampling method. C) Same as in A & B but employing our new sampling method introduced in **Methods**. (A,B,C) show the bins thresholded at 100 unique InChiKey pairs. Color represents the log base 10 count of structure pairs. (D,E,F) show the unthresholded log count of pairs in each bin.


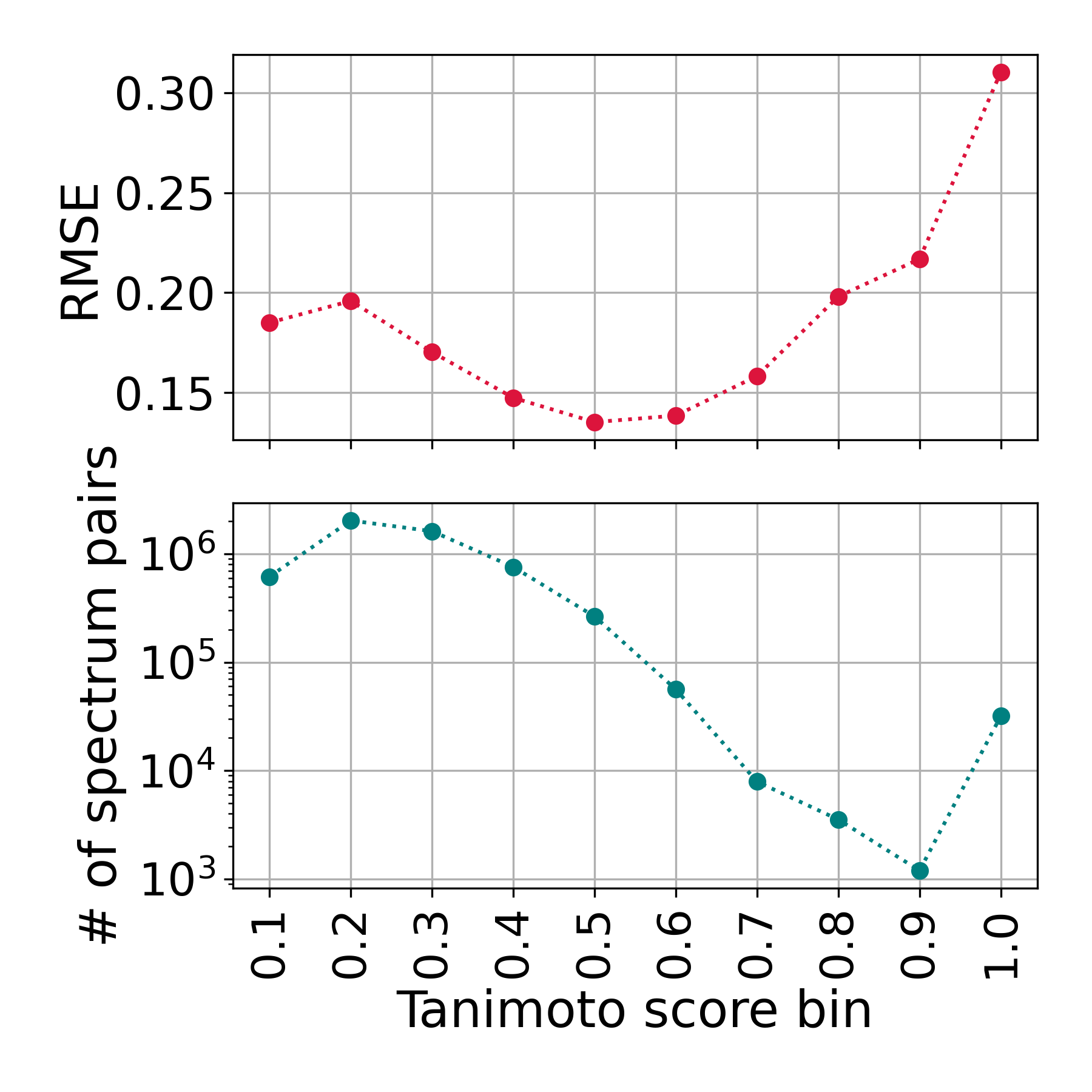

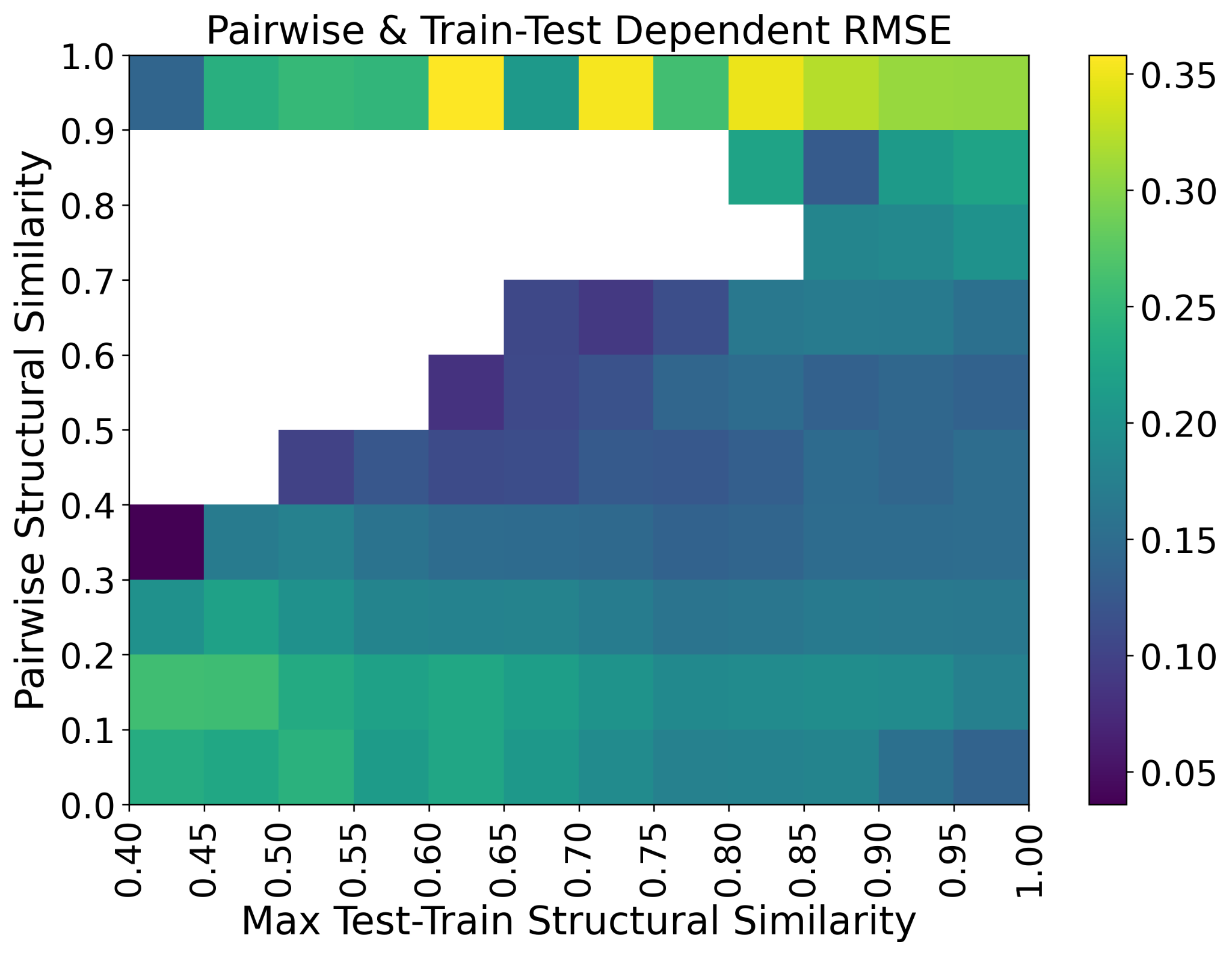


A

B

C

**SI Figure 3 – Reproduction of Original Results.** A) RMSE binned by ground-truth Tanimoto similarity on a 500 InChiKey test set generated from the same data used in this manuscript. Results across bins are comparable to (Huber et al. 2021). As a consequence, we do not further adjust MS2DeepScore hyperparameters in this work. B) RMSE binned by ground truth pairwise similarity and train-test similarity. Blank cells indicate no pairs. C) Binned count of pairwise similarities within the 500 InChiKey test set. X-tick values represent the exclusive upper end of the pairwise similarity bin (e.g., 0.4 is the [0.3, 0.4) bin), with the exception of 1.0 which is inclusive.


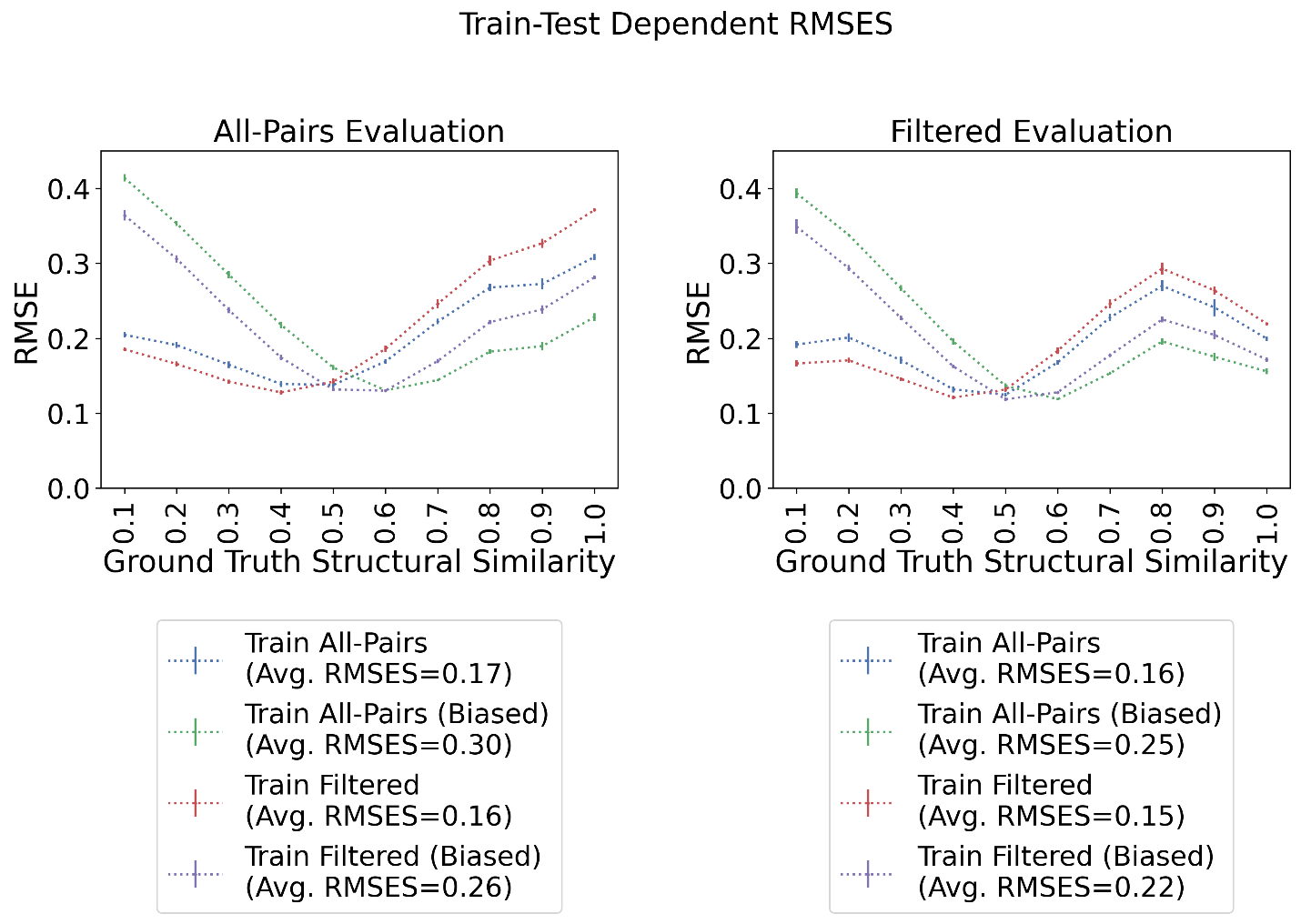


A

B

**SI Figure 4 – Standard Deviation of Performance Relative to Ground Truth Similarity** A) Root Mean Squared Error (RMSE) comparison between All-Pairs, Filtered, unbiased, and biased models on All-Pairs test data across 10 ground truth similarity bins. B) RMSE comparison on the Filtered test dataset. Error bars show standard deviation across 4 random seeds.


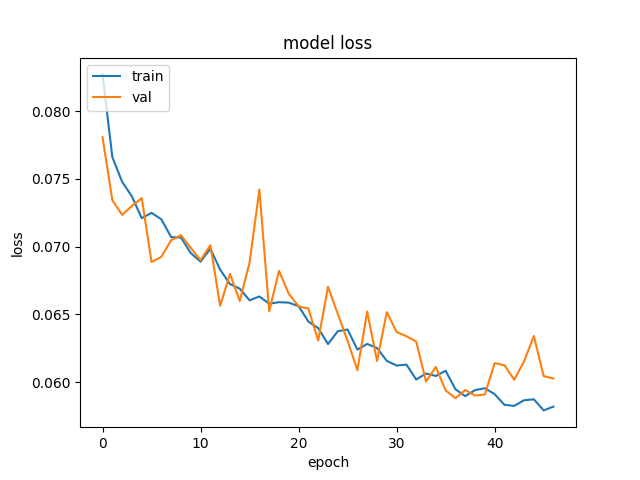

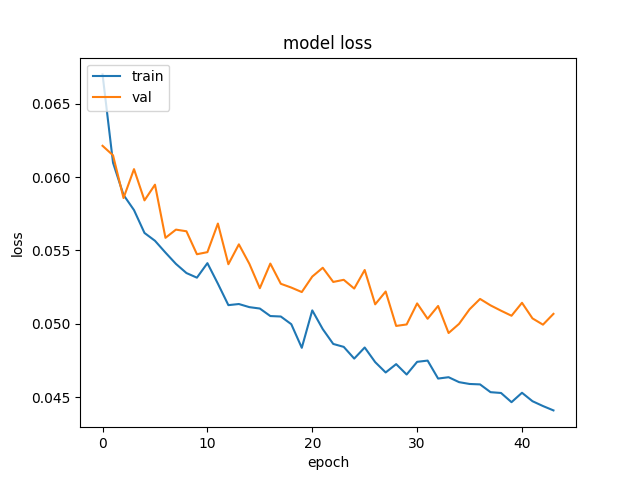


B

A

**SI Figure 5 – Mean Squared Error (MSE) Loss Curves** Loss reported over 150 epochs of training for the All-Pairs and Filtered models reported within the main text with early stopping. A) MS2DeepScore training on the All-Pairs and B) the Filtered training/validation sets.


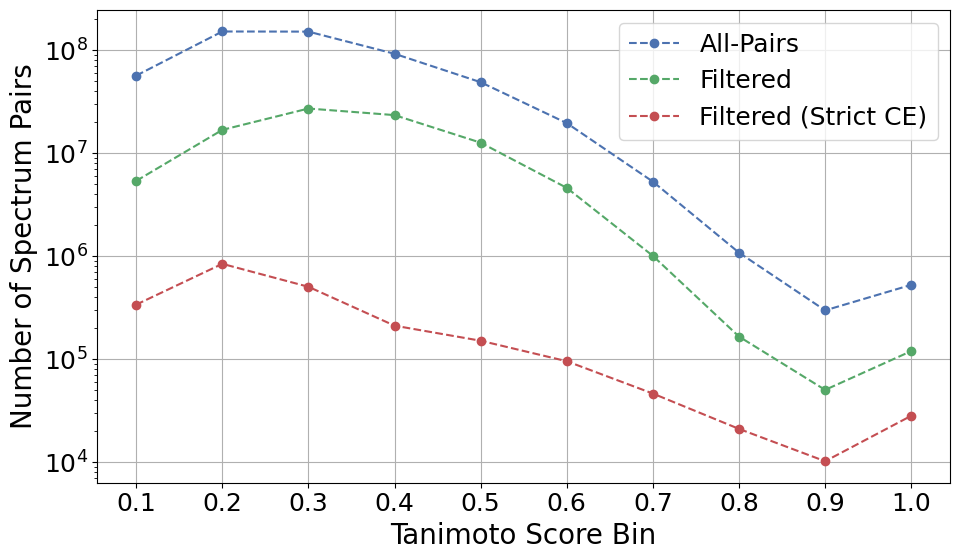


**SI Figure** **6 – Pairwise Similarity Distribution in Evaluation Data.** The distribution of spectrum pairs in the All-Pairs, Filtered, and Filtered (Strict-CE) evaluation set. The Strict-CE evaluation set requires both spectra in the pair to be annotated with collision energies.

##


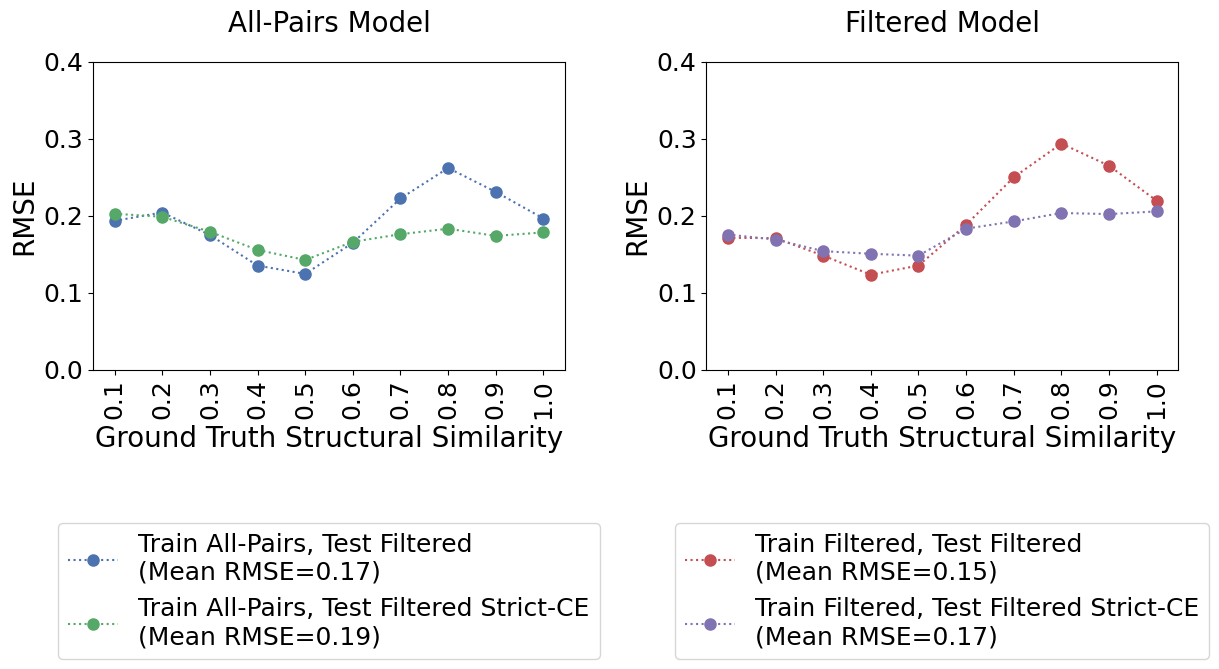


A

B

**SI Figure 7 – Comparison of Performance on Filtered Data with and Without Strict Collision Energy Criteria** A) Evaluation of the All-Pairs model on the standard Filtered and strict CE Filtered evaluation set. B) Evaluation of the Filtered model on the standard Filtered and strict CE Filtered evaluation set. Within the strict-CE filtered dataset CE is required for both spectra in all pairs.

##

**SI Note 1 - Strict Collision Energy Evaluation**

Due to limitations in the provenance data, collision energies (CEs) were only available for 9,530/32,555 of test spectra (29.27%), resulting in a test dataset with 4,511,562 spectrum pairs (from 1,636 structures) after applying all filters and requiring collision energies. Therefore, it is difficult to report granular metrics for such a subset. Instead, in the interest of further investigating the effect of collision energies on high-pairwise similarity pairs, we only evaluate RMSE across pairwise similarity bins (**Fig. 7**).

The indicated performance improvement between evaluation on the Filtered and strict-CE Filtered data sets is most pronounced in the range of [0.6. 0.9). For the [0.7, 0.8) bin, error reduces from 0.2619 to 0.1830 and 0.2937 to 0.2035 for the All-Pairs and Filtered models respectively. Notably, the reduction in RMSE for pairs of spectra with a ground-truth structural similarity greater than 0.90 is limited: 0.1963 to 0.1783 for the All-Pairs model and 0.2194 to 0.2055 for the Filtered model evaluated on the Filtered and strict-CE test sets respectively.

##

**SI Note 2 - Limitations of RMSE as a Metric**

The discrepancy in model performance on high-similarity pairs (**Fig. 3**) underscores the limitations of averaged metrics such as RMSE and MAE as comprehensive metrics. Primarily, this is a result of the imbalance of pairwise similarities within the test dataset where lower pairwise similarities are overrepresented while higher similarities are underrepresented. The distribution of pairwise similarities within the All-Pairs and Filtered test set is included in Fig. . In future work, aggregated metrics should be reported over equal-width bins to mitigate this bias. For example, the model trained and evaluated on the All-Pair dataset reports a RMSE of 0.2031 while the model trained on the Filtered dataset achieves a global average RMSE of 0.1688. However, despite the lower average RMSE, the model trained on Filtered data significantly underperforms on high pairwise similarities (**See Results: Training on Filtered Data, Fig. 3A**).

**SI Figure 8 – Tanimoto Similarity for Top Predictions** The maximum Tanimoto similarity of the top k compounds with highest predicted similarity. Each data point represents the average across four training seeds. Error bars represent standard deviation with N-1 degrees of freedom. A) The maximum Tanimoto similarity of the top k compounds with highest predicted similarity averaged across test data points. The optimal structure similarity (Theoretical Maximum) is also included which denotes an upper bound on performance. Note that the error bars in this plot are small and are not clearly discernible. B) Tanimoto similarity excluding any compounds with an identical planar 2D structure. On average, all variants of the MS2DeepScore model retrieve data points with comparable Tanimoto similarity to the modified cosine similarity. Note that the error bars in this plot are small and are not clearly discernible.


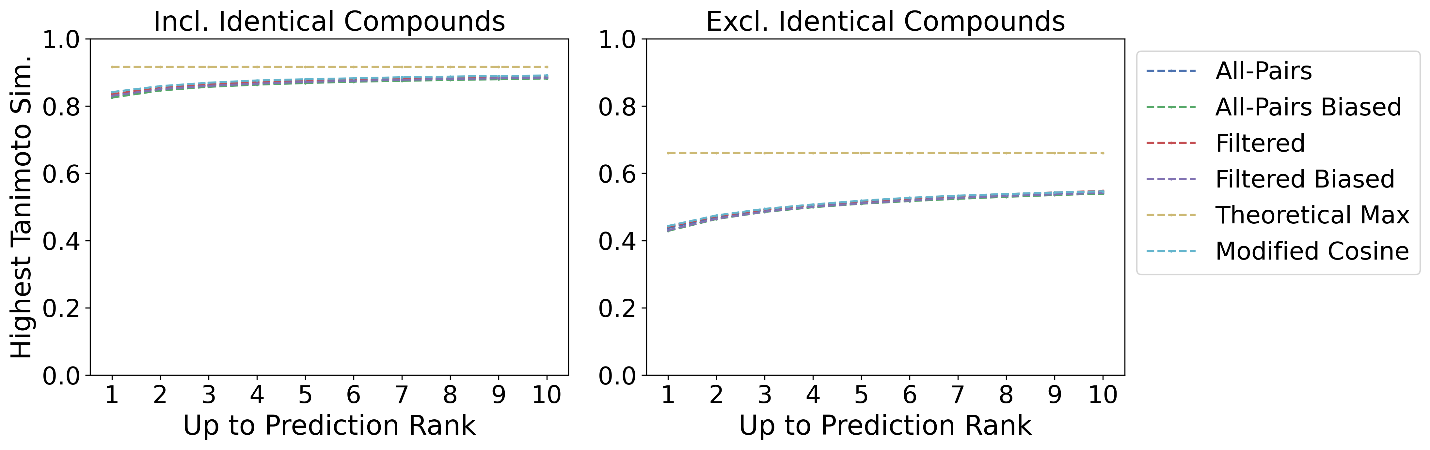


A

B


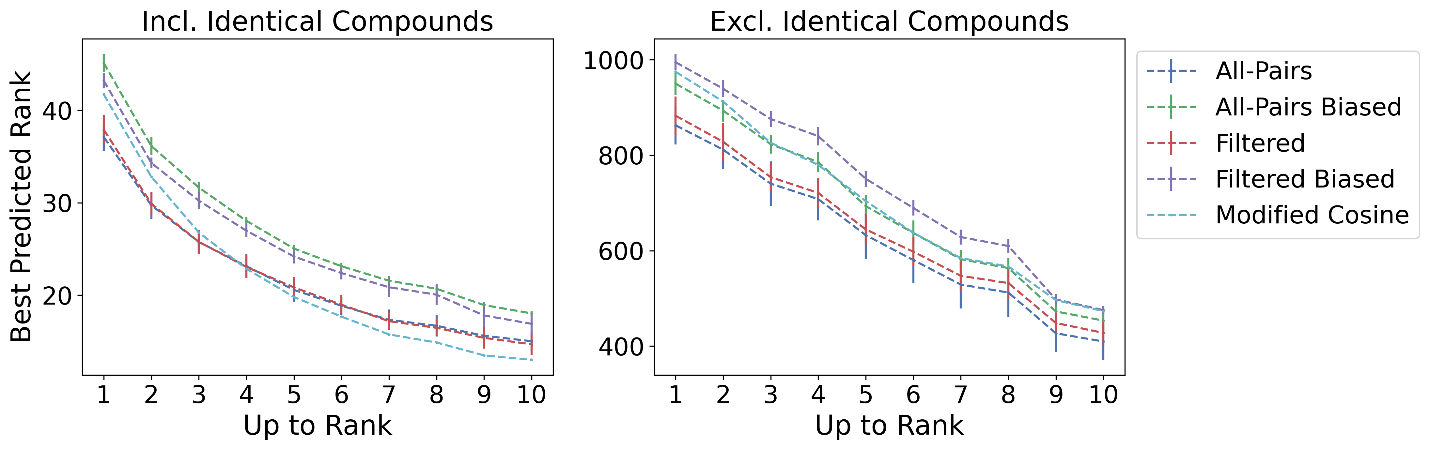


A

B

**SI Figure 9 – Top Rank Scores** The best predicted rank for the k when including identical compounds (A) and excluding identical compounds (B). Each data point represents the average across four training seeds. Error bars (vertical lines) represent standard deviation with N-1 degrees of freedom. A) The best predicted rank for the k most similar structures, averaged across all test pairs. The “All-Pairs model” and “Filtered model” retrieve the highest similarity structures up to ground-truth rank k=3, at which point it’s comparable to modified cosine. B) The best predicted rank for the k most similar structures, excluding identical structures. The All-Pairs model outperforms all other methods at any maximum k value.


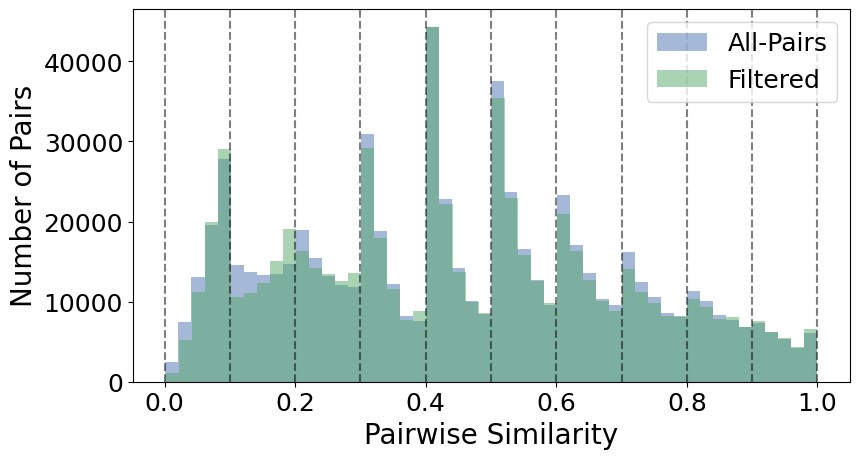

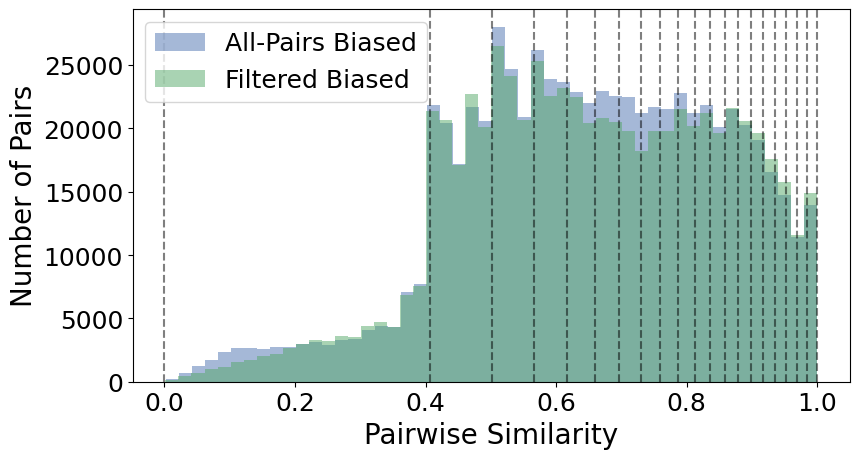


A

B

**SI Figure 10 – Distribution of Pairwise Similarities in Training Data**. A) The distribution of pairwise similarities for unbiased All-Pairs and Filtered datasets. B) The distribution of pairwise similarities of the All-Pairs and Filtered data sets when using the biased sampling method introduced in **Methods: Biased Training of MS2DeepScore**. Within each bin, the distribution of pairs biases towards the underlying distribution. As a result, neither perfectly conforms to the target distribution. Further, if an InChiKey fails to produce a match within the target bin, the bin is iteratively widened allowing the sampled distribution to stray from the target distribution. Plots show 20 epochs of training data.


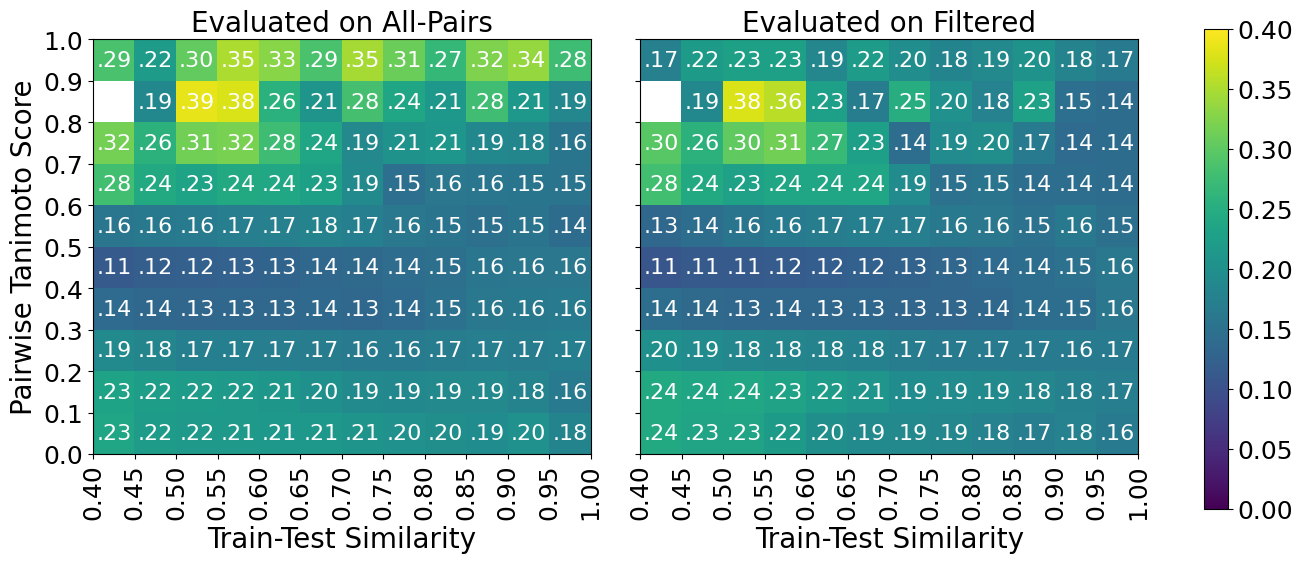

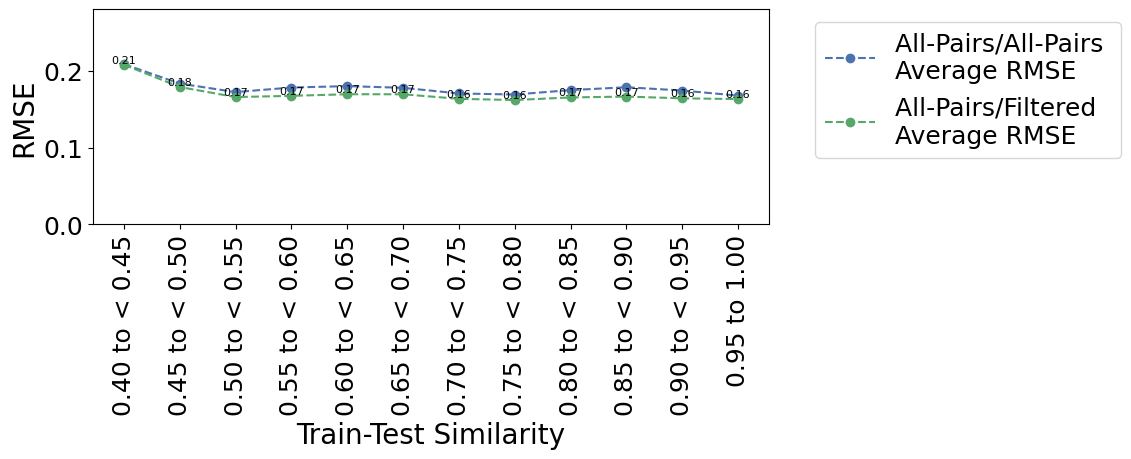


A

B

C

**SI Figure 11 – RMSE by Pairwise Similarity and Train-Test Similarity** A) The RMSE of the All-Pairs model on the All-Pairs test set binned by test-train similarity and pairwise similarity. B) RMSE of the All-Pairs model on the Filtered test set binned by test-train similarity and pairwise similarity. RMSE tends to decrease as train-test similarity increases (left to right). C) The marginal RMSE as distance to the training set decreased for the All-Pairs model on the All-Pairs and Filtered evaluation sets. RMSE decreases from the least to most similar data points (0.21 to 0.17 and 0.21 to 0.16 for the All-Pairs and Filtered datasets respectively). This trend is more apparent in higher (>0.6) and lower (<0.3) pairwise ground-truth structural similarities than it is in the (0.2 0.6) range.


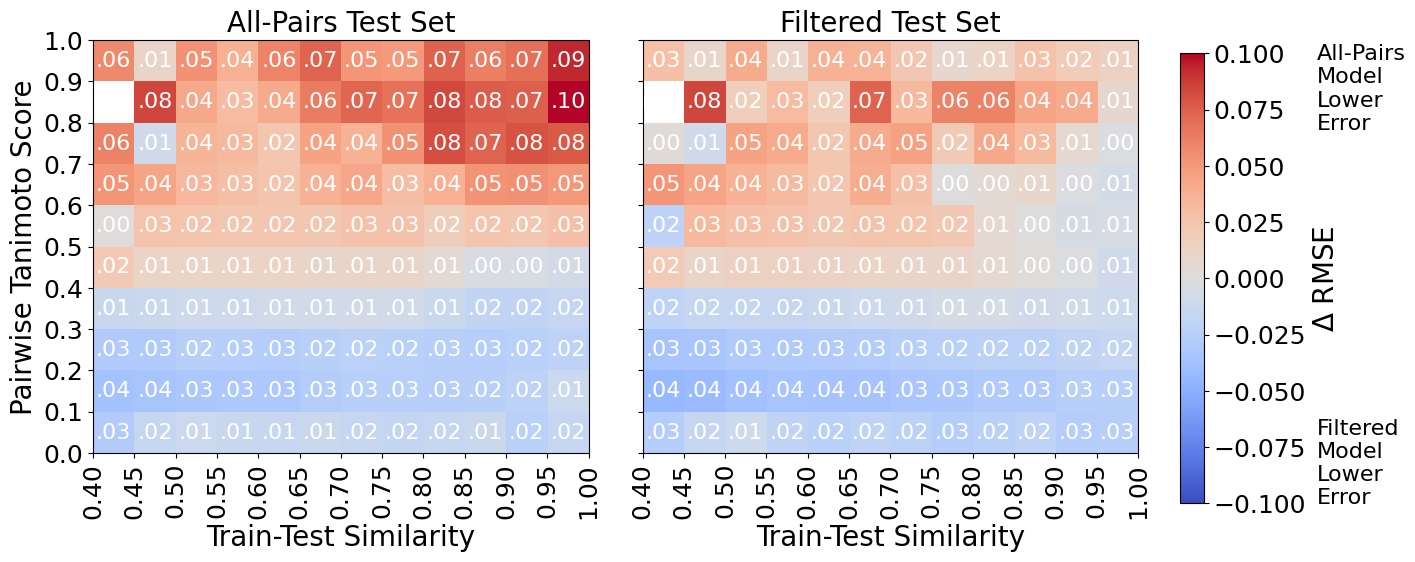


A

B

**SI Figure 12 – Comparison of Filtered and All-Pairs Models**. A) The RMSE of the All-Pairs model subtracted from the error of the Filtered model on the All-Pairs evaluation set. B) The RMSE of the All-Pairs model subtracted from the error of the Filtered model on the Filtered evaluation set. Negative numbers (shown in blue) show areas where the Filtered model performs better, while positive numbers (shown in red) show areas where the All-Pairs model performs better. The model trained on All-Pairs consistently outperforms the model trained on Filtered data across all test-train similarity bins for pairwise structural similarities greater than 0.50. In comparison to the Filtered set, a larger distance between models is observed on the All-Pairs set for spectra corresponding to high pairwise similarity structures.

| Training Set | Evaluation Set | Biased? | Test RMSE | Test RMSE  (Pairwise Sim.  > 0.6) |
| --- | --- | --- | --- | --- |
| All-Pairs | All-Pairs | N | 0.1743 | 0.2629 |
| All-Pairs | All-Pairs | Y | 0.2318 | 0.1823 |
| All-Pairs | Filtered | N | 0.1670 | 0.2278 |
| All-Pairs | Filtered | Y | 0.2135 | 0.1663 |

**SI Table 1 – RMSE of All-Pairs Model**. The RMSE for the All-Pairs model on all training and evaluation conditions. The model achieves the best overall RMSE for the All-Pairs and Filtered datasets when trained on unbiased data. However, the model achieves the best performance for structurally related pairs (pairwise similarity > 0.6) on the Filtered dataset with biased training data. For the pairwise similarity > 0.6, we bin data points into four train-test similarity bins and equally weigh the average.

|  | | | Top Candidate Similarity (Standard Deviation) | | | | | |
| --- | --- | --- | --- | --- | --- | --- | --- | --- |
|  |  |  | k=1 | k=3 | k=10 | k=1 | k=3 | k=10 |
| Training Set | Test Set | Biased? | Incl. Ident. | Incl. Ident. | Incl. Ident. | Excl. Ident. | Excl. Ident. | Excl. Ident. |
| All-Pairs | All-Pairs | N | 0.9138 (0.0028) | 0.9250  (0.0021) | 0.9493 (0.0021) | 0.4755 (0.0009) | 0.4967 (0.0017) | 0.5537 (0.0014) |
| All-Pairs | All-Pairs | Y | 0.9121 (0.0044) | 0.9238 (0.0036) | 0.9499 (0.0023) | 0.4663 (0.0007) | 0.4879 (0.0014) | 0.5470  (0.0002) |
| All-Pairs | Filtered | N | 0.8505 (0.0034) | 0.8619 (0.0043) | 0.8847 (0.0031) | 0.4703  (0.0069) | 0.4909  (0.0073) | 0.5456  (0.0051) |
| All-Pairs | Filtered | Y | 0.8464 (0.0031) | 0.8573 (0.0039) | 0.8816  (0.0032) | 0.4641  (0.0016) | 0.4853  (0.0026) | 0.5396  (0.5395) |

**SI Table 2 – Retrieval Metrics for the All-Pairs Model.** The *Top Candidate Similarity* of biased and unbiased models on the All-Pairs and Filtered test sets. A higher score reflects better performance. Across all conditions, evaluation on the Filtered set demonstrates better metrics. Across all test sets and criteria, the models trained on biased data outperformed models trained on unbiased data. Metrics reported are mean and standard deviation across four random seeds. Standard deviation is calculated with N-1 degrees of freedom.

|  | | | Top Rank (Standard Deviation) | | | | | |
| --- | --- | --- | --- | --- | --- | --- | --- | --- |
|  |  |  | k=1 | k=3 | k=10 | k=1 | k=3 | k=10 |
| Training Set | Test Set | Biased? | Incl. Ident. | Incl. Ident. | Incl. Ident. | Excl. Ident. | Excl. Ident. | Excl. Ident. |
| All-Pairs | All-Pairs | N | 169.6724 (7.05) | 117.6429 (6.25) | 78.6387 (4.78) | 3299.0360 (70.86) | 2200.7026 (42.88) | 1237.4284 (56.98) |
| All-Pairs | All-Pairs | Y | 197.7982 (2.52) | 137.9874 (3.00) | 86.9086 (2.38) | 3975.2109 (113.09) | 2769.78753 (26.27) | 1501.2471 (31.00) |
| All-Pairs | Filtered | N | 37.1079 (1.52) | 25.7518 (1.31) | 13.8622 (1.05) | 862.5562 (40.01) | 740.3154 (46.51) | 409.6317 (38.28) |
| All-Pairs | Filtered | Y | 45.1207 (0.95) | 31.636 (0.66) | 18.0241 (0.26) | 949.8339 (23.30) | 822.9592 (19.76) | 453.3658 (23.53) |

**SI Table 3 – Ranking Metrics for the All-Pairs Model.** The *Top Rank* metric of biased and unbiased models on the All-Pairs and Filtered test sets. The score reflects the lowest rank of a structure within the kth most similar structures; a lower score is better. When Identical compounds are included in retrieval all models are capable of ranking one of the top 10 most similar structures within the top 20 candidates within the Filtered test set. However, when identical compounds are excluded, all models fail to rank the top 10 most similar structures within the top 300 on the filtered test set. Metrics reported are mean and standard deviation across four random seems. Standard deviation is calculated with N-1 degrees of freedom.
